# Stack-Engineered Mode Selection in PtMn/(Co/Pd)_n_ Multilayers Enables Deterministic Analog Spin–Orbit Torque Synapses

**DOI:** 10.1021/acsami.5c22532

**Published:** 2026-03-11

**Authors:** Abhijeet Ranjan, Tamkeen Farooq, Chong-Chi Chi, Chao-Chin Wang, Yu-Lon Lin, Rudis Ismael Salinas Padilla, Ying-Hung Li, Yuan-Chieh Tseng, Chih-Hao Lee, Ming-Yen Lu, Rahul Mishra, Chih-Huang Lai

**Affiliations:** † Department of Materials Science and Engineering, 34881 National Tsing Hua University, Hsinchu 30013, Taiwan; ‡ Centre for Applied Research in Electronics, 28817 Indian Institute of Technology Delhi, New Delhi 110016, India; § Instrumentation Center, 34881 National Tsing Hua University, Hsinchu 30013, Taiwan; ∥ Department of Engineering and System Science, 34881 National Tsing Hua University, Hsinchu 30013, Taiwan; ⊥ Department of Materials Science & Engineering, 34914 National Yang-Ming Chiao-Tung University, Hsinchu 30010, Taiwan; # Institute of Space Systems Engineering, National Yang-Ming Chiao-Tung University, Hsinchu 30010, Taiwan; ∇ College of Semiconductor Research, National Tsing Hua University, Hsinchu 30013, Taiwan

**Keywords:** spin−orbit torque, binary switching, multilevel switching, neuromorphic computing, artificial synapses, artificial neural networks

## Abstract

Spin–orbit-torque (SOT) devices that support both binary and analog switching can bridge spintronic memory and neuromorphic computing, provided the switching mode can be deliberately assigned. Here, we demonstrate that in PtMn/(Co/Pd)­n multilayers, the Co/Pd repeat number, n_Co/Pd_, serves as a material parameter that determines the reversal mechanism and switching mode. For n_Co/Pd_ ≤ 7, magnetization reversal is governed by nucleation followed by domain-wall propagation, resulting in binary switching that can undergo a transition to analog behavior through electrical conditioning. For n_Co/Pd_ ≥ 8, increased structural modulation in the as-deposited state suppresses domain-wall propagation and stabilizes nucleation-dominated analog switching without additional processing. Applying controlled current conditioning to these high-n_Co/Pd_ stacks produces a hybrid state with smoother long-term potentiation and depression, an expanded number of intermediate states, and neuromorphic classification accuracy exceeding 97%. These results establish stack design and hybrid tuning as scalable strategies for energy-efficient analog SOT synapses in neuromorphic hardware.

## Graphical abstract

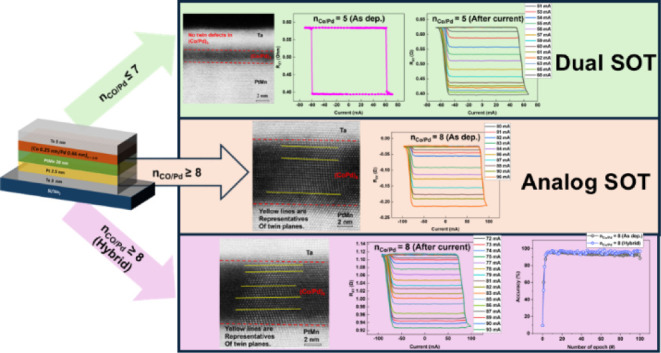

## 1 Introduction

Neuromorphic computing aims to overcome the energy and data-transfer bottlenecks of conventional von Neumann architectures by colocating memory and computation. Spintronic devices are attractive candidates because they offer nonvolatility, low-power operation, high endurance, and CMOS compatibility.^1–10^ Among them, spin–orbit torque (SOT) devices enable efficient electrical control of magnetization using in-plane current, supporting fast and reliable write operations.^11–20^ Depending on the magnetization-reversal pathway, SOT devices can exhibit either binary or analog switching.^2–5,11–15^ Binary switching is well-suited for digital storage and neuron-like functionalities, whereas analog switching enables gradual, incremental weight updates required for synaptic learning.^21–34^ Consequently, scalable neuromorphic hardware requires strategies that can deliberately assign and, ideally, tune the switching mode within a unified materials platform.^1–5^

Recent studies have demonstrated that the SOT switching characteristics significantly impact neuromorphic performance. For example, Guo et al. achieved multilevel states in Pt/Co/Ta heterostructures and demonstrated a classification accuracy of 93.38% in a convolutional neural network task.^26^ Yadav et al. used gradient multilayers in Pt/Co-based SOT devices to access numerous stable states while maintaining thermal stability, achieving 92.2% accuracy.^27^ Topological-insulator-based SOT neurons and synapses have also achieved ultralow switching current densities with high recognition performance.^28^ These results emphasize that neuromorphic performance is tightly linked to how precisely the magnetic state can be incrementally and reproducibly updated.

A key materials question is how to deliberately favor nucleation-dominated analog switching over domain-wall-propagation-driven binary switching. Wan et al. induced a gradual switching in Pt/Co/MgO by focused ion-beam illumination, which locally softens the magnetic properties, thereby increasing the number of nucleation events and intermediate states.^29^ In a complementary direction, Zhou et al. reported field-free, nucleation-dominated SOT switching in an L1_1_-CuPt/CoPt bilayer and used the resulting intermediate states to implement a sigmoidal spintronic neuron for MNIST classification; they attributed the nucleation-dominated behavior at H_x_ = 0 to DMI-fixed Néel-wall chirality that suppresses deterministic domain-wall propagation and to spatially distributed nucleation barriers E_n_, such that switching progresses mainly through additional nucleation rather than propagation.^30^ These works highlight that engineering microstructure and local energy landscapes can promote analog-like SOT responses, but they typically rely on postfabrication modification or specific material systems and do not directly provide a fabrication-time parameter to deterministically assign the switching mode.^2,5,9,25,31–33^

Related approaches based on stacking periodicity and AFM/FM coupling further illustrate the role of microstructure and domain statistics. In Co/Pt multilayers, multilevel states can be achieved through multidomain formation where the final magnetization can be programmed by pulse conditions in an initialization-free manner.^5^ In PtMn/[Co/Ni], analog-like (memristive) switching has been linked to exchange bias and grain-scale variations that inhibit domain-wall propagation, yielding stable intermediate multilevel states.^2,9,25^ In addition, electrical or thermal treatments can modify defect distributions and convert binary to analog switching in PtMn/(Co/Pd)_4_,^34^ but such postgrowth conditioning is applied only after fabrication and can introduce variability across devices. For synaptic functionality, it is also essential to maintain a large number of stable intermediate states while achieving linear and symmetric long-term potentiation and depression.^1–5,31–33^

Here, we demonstrate that the Co/Pd repeat number, n_Co/Pd_, in PtMn/(Co/Pd)_n_ multilayers provides a materials-level, fabrication-time structural parameter that deterministically selects the SOT switching mode. By tuning n_Co/Pd_ from 2 to 9, we show that devices with n_Co/Pd_ ≤ 7 exhibit binary switching in the as-deposited state that can be converted to analog behavior through controlled electrical conditioning, whereas devices with n_Co/Pd_ ≥ 8 display analog switching already in the as-deposited state. Transmission electron microscopy and Kerr imaging reveal that increasing n_Co/Pd_ systematically modifies the multilayer structural modulation and the domain-wall pinning environment, correlating with a crossover from domain-wall-propagation-driven reversal to nucleation-dominated analog switching. Furthermore, mild current conditioning of high-n_Co/Pd_ stacks produces a hybrid state with smoother and more symmetric synaptic updates, an expanded number of intermediate states, and recognition accuracy exceeding 97% on the MNIST data set. These results establish stack design and hybrid tuning as scalable routes to thermally stable and energy-efficient analog SOT synapses for neuromorphic computing.

## 2 Experimental and Methods

Si/SiO_2_//Ta­(3)/Pt­(2.5)/Pt_50_Mn_50_(20)/[Co­(0.25)/Pd­(0.66)]_n_/Ta­(5) (thickness in nm) multilayers were fabricated on Si/SiO_2_ substrates using magnetron sputtering. The stack consisted of Ta (3 nm) for adhesion and Pt (2.5 nm) to promote Pt_50_Mn_50_ (111) texture. The Pt_50_Mn_50_ (20 nm) and Ta (5 nm) layers serve as the spin current source; the ferromagnetic layer is composed of [Co­(0.25 nm)/Pd­(0.66 nm)]_n_ with n_Co/Pd_ = 2–9. Ta (5 nm) is also used for the capping. Film thicknesses were verified by atomic force microscopy (AFM). All deposits were performed in an Ar atmosphere at room temperature (base pressure <2 × 10^–7^ Torr). Magnetic properties were measured using a vibrating sample magnetometer (VSM) and a superconducting quantum interference device (SQUID). Hall cross devices (10 μm × 40 μm) were patterned by photolithography and etched using Ar ion milling down to the SiO_2_ substrate. After the device fabrication, all the SOT measurements were performed using 300 μs long pulses unless stated otherwise. High-resolution transmission electron microscopy (HRTEM) imaging was performed with a spherical-aberration-corrected field-emission electron microscope (JEM ARM-200FTH) for structural analysis.

## 3 Results and Discussion

### 3.1 Property Evolution with Co/Pd Repeat Number

Figure 1a demonstrates the schematic of the heterostructure used in this study. The magnetic properties of each of these as-deposited heterostructures are shown in Figure 1b–1d. As shown in Figure 1b, each loop exhibits robust perpendicular magnetic anisotropy, as evidenced by M_R_/M_S_ = 1 in each case. As n_Co/Pd_ increases, net magnetization increases. The saturation magnetization was larger than that of bulk Co due to the induced magnetization in the Pd layers. The variation of coercivity (H_C_) with n_Co/Pd_ is shown in Figure 1c; it increases linearly up to n_Co/Pd_ = 6, and then increases gradually. The anisotropy field (H_K_) also scales nearly linearly with n_Co/Pd_, as shown in Figure 1d, because the number of Co/Pd interfaces increases as n_Co/Pd_ increases, consistent with the previous report.^35^ Note that all the films are as-deposited films without any postannealing, so we do not build any exchange bias along any direction. The increase in coercivity can be attributed to either the increase in uniaxial anisotropy or the enhanced wall-pinning effect resulting from the accumulation of structural defects at the interfaces with increasing Co/Pd repeats.^35^ Figure 1e shows an optical micrograph of the fabricated Hall cross devices along with the schematic of the measurement setup used for SOT characterization, where the anomalous Hall effect (AHE) serves as the readout signal. The micron-scale Hall cross structures exhibit robust perpendicular magnetic anisotropy (PMA), as confirmed by the square-shaped field-dependent AHE loops presented in Figure 1f.

**Figure 1:**
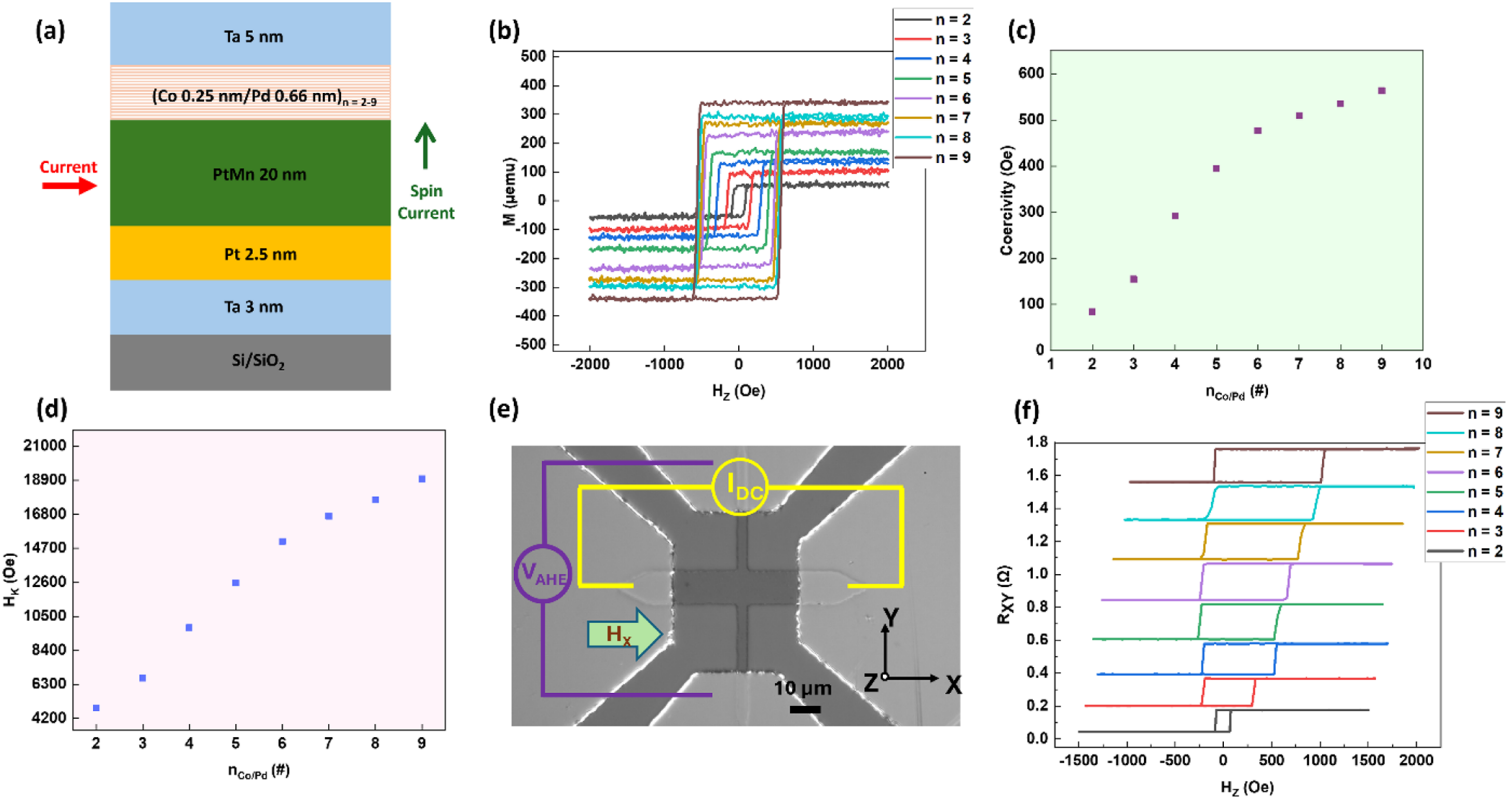
Magnetic Properties of PtMn/(Co/Pd)_n=2–9_. (a) The schematics of the heterostructures used in this study, (b) Out-of-plane M–H loops showing robust perpendicular magnetic anisotropy (PMA) and high squareness for all n_Co/Pd_, (c) Coercivity H_C_ versus Co/Pd repeat number n_Co/Pd_, (d) Anisotropy field H_K_ versus Co/Pd repeat number n_Co/Pd_, (e) Optical micrograph of a fabricated Hall-cross device and the SOT measurement schematic using the anomalous Hall effect (AHE) as the readout. (f) Field-swept AHE loops for each device, confirming that PMA is preserved after device fabrication.

### 3.2 Switching Pathways: Nucleation vs Domain-Wall Propagation

We performed SOT switching measurements for all devices with n_Co/Pd_ = 2–9, applying current pulses of 300 μs width while incrementally increasing the current amplitude and reading the magnetization state after each pulse. Also note that an in-plane field H_X_ = 40 Oe was applied during the SOT switching throughout. For n_Co/Pd_ = 2–7, the devices reproducibly exhibited dual SOT switching behavior, consistent with previously reported results^34^ for n_Co/Pd_ = 4. A representative example for n_Co/Pd_ = 5 is shown in Figure 2a–2d. As shown in Figure 2a, reversible magnetization switching between up and down states occurs at ±70 mA under a modest in-plane assist field H_x_ = 40 Oe. The switching polarity corresponds to the spin Hall effect (SHE), which is generated by the bottom PtMn layer and/or top Ta layer, with negligible spin contribution from the thin Pd spacer. The sharp, binary nature of the loop is characteristic of domain-wall nucleation and propagation, which drives the reversal in conventional heavy-metal/ferromagnet systems. The SOT efficiency and DMI field were measured by using a loop shift method for a representative device n_Co/Pd_ = 5. The efficiency value is 0.235, slightly higher or comparable to those reported for PtMn earlier,^36,37^ suggesting that the top Ta layer may partially contribute to the spin current source. The DMI field was found to be approximately 50 Oe, which scales well with the low H_X_ required for SOT switching in our system. The contribution of PtMn and Ta to SOT switching is discussed in detail in the Supporting Information S1.

**Figure 2:**
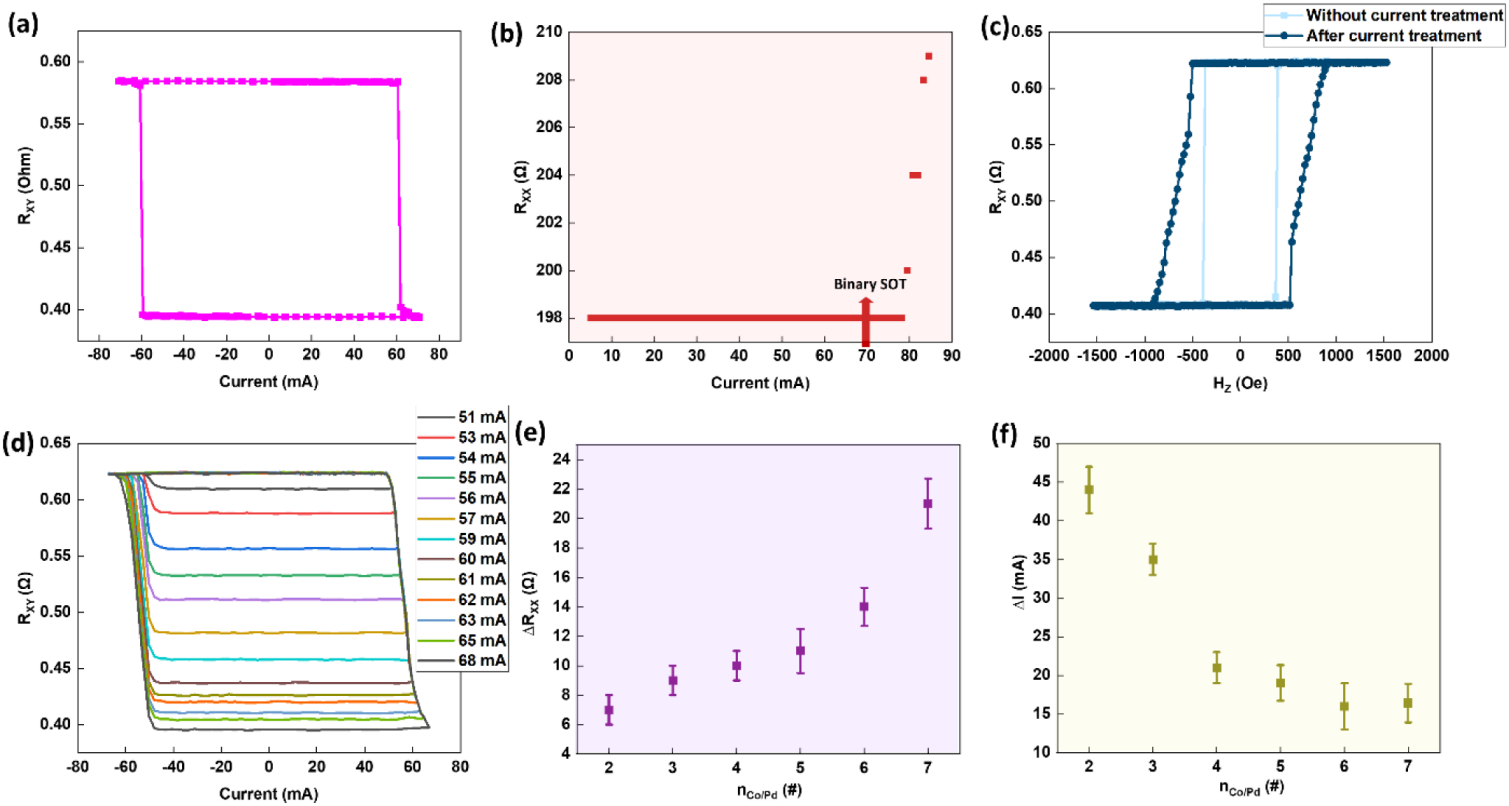
Dual SOT switching modes observed in PtMn/(Co/Pd)_n=2–7_; (a–d) Representative dual SOT mode for n_Co/Pd_ = 5. (a) Current driven binary SOT (±70 mA) switching under H_X_ = 40 Oe, (b) Change of current channel resistance (R_XX_) with the input current along the current channel (R_XX_), (c) AHE loop by field switching before and after current treatment, (d) Analog SOT switching under H_X_ = 40 Oe after large current treatment (85 mA), (e) Variations of resistance along the current channel (R_XX_) for the device with n_Co/Pd_ = 2– 7 after a large current treatment, and (f) The difference between the current required to have binary SOT switching and the current required to cause the transition from the binary SOT mode to multilevel SOT mode (ΔI) for n_Co/Pd_ = 2–7. The pulse width is 300 μs for each pulse during SOT switching.

Upon increasing the current amplitude while maintaining the 300 μs pulse width, a transition is observed near 85 mA, as evidenced by an increase in the longitudinal resistance R_xx_ (Figure 2b). The transition current magnitude is defined as |I_transition_|. Comparing the field-swept AHE loops before and after this high-current treatment (Figure 2c) reveals enhanced coercivity and a sheared, slanted loop shape, signatures of structural modification and increased domain wall pinning. Following this irreversible transition, the magnetization switches gradually, producing multilevel, analog SOT characteristics as shown in Figure 2d. The same dual SOT behavior was also observed using shorter pulses (10 μs) and is shown in Supporting Information S2 for representative devices.

The origin of this transition mirrors earlier findings for n_Co/Pd_ = 4: high-current treatment induces twin formation in Co/Pd and PtMn layers, creating pinning sites that hinder domain-wall propagation.^34^ Consequently, magnetization reversal becomes nucleation-dominated, producing stable analog switching. Before the current treatment, the absence of such twin defects allows efficient domain-wall motion, resulting in binary switching.^34^ Figures 2e summarize the change of the longitudinal resistance change (ΔR_xx_(n)) for each n_Co/Pd_ = 2–7 where ΔR_XX_(n) = R_xx,after_ – R_xx,before_; R_xx,before_ is measured in the pristine binary regime using a small DC read current (well below the switching threshold), and R_xx,after_ is measured after the irreversible transition (i.e., after applying |I_transition_|) under the same readout conditions while Figure 2f summarizes the difference between the current required to have binary SOT switching and the current required to cause the transition from the binary mode to multilevel SOT mode (ΔI), where ΔI is defined as ΔI­(n) = |I_transition_(n)| – |I_binary_(n)| for each n_Co/Pd_ = 2–7. This quantity represents a practical current “guard band” separating binary-mode operation from the onset of the analog regime under the same pulse width. The reported values of ΔR_XX_ and ΔI­(n) in Figure 2e and 2f are averaged across multiple devices and are plotted with corresponding error bars.

For n_Co/Pd_ = 2–7, a larger ΔI provides a wider guard band that maintains binary stability against write-current fluctuations, whereas a smaller ΔI implies a more fragile binary state. Reduction in ΔI with increasing n_Co/Pd_ directly follows the rise in twin density observed in HRTEM images (will be shown below in Figure 5): as n_Co/Pd_ increases, the higher density of twin-induced pinning centers suppresses long-range domain-wall propagation, lowering the current required to enter the nucleation-dominated (analog) regime. The corresponding change in device resistance (ΔR_XX_(n)) follows a similar trend. The pristine resistance R_XX_ decreases with n_Co/Pd_ due to increased film thickness. In contrast, current treatment increases R_XX_ via twin formation, intermixing, and interface shear, which introduce additional scattering. For n_Co/Pd_ = 2–7, ΔR_XX_(n) increases monotonically (∼6 Ω to ∼22 Ω), reflecting greater available Co/Pd interface area for defect formation. These results confirm that dual SOT modes persist up to n_Co/Pd_ = 7 with the same microscopic mechanism as in previous work,^34^ while also demonstrating the tunability of SOT behavior through controlled current excitation and selecting a device that can both protect the binary mode against maximum fluctuation and provide the best analog behavior. This provides a powerful route to engineer reconfigurable spintronic devices capable of toggling between binary (memory-like or neuronal) and analog (synaptic) operations within the same structure, thereby enhancing flexibility for neuromorphic computing architectures.

### 3.3 Deterministic Mode Selection via Co/Pd Repeat Number

With increasing the number of repeat (n_Co/Pd_ = 8 and 9), we observed purely multilevel (analog) SOT switching even in the as-deposited state, without requiring any high-current or thermal treatment. Figure 3a and 3b show representative current-induced switching curves for n_Co/Pd_ = 8 and n_Co/Pd_ = 9, respectively, both exhibiting gradual, nonabrupt resistance evolution characteristic of analog behavior. The in situ Kerr image in Figure 3c for the as-fabricated device with n_Co/Pd_ = 8 further confirms that magnetization reversal proceeds via nucleation-dominated switching and/or suppressed propagation, leading to analog SOT switching. The reversal dynamics for the as-fabricated device with n_Co/Pd_ = 8 is distinct from those observed in the as-fabricated device with n_Co/Pd_ = 5, shown in Figure 3d, where we can clearly see switching takes place via nucleation of an opposite domain, which propagates very fast upon increasing current. This contrasts sharply with the dual-mode behavior observed for n_Co/Pd_ = 2–7, where devices initially exhibit sharp binary SOT switching but transition to analog behavior only after high-current treatment.

**Figure 3:**
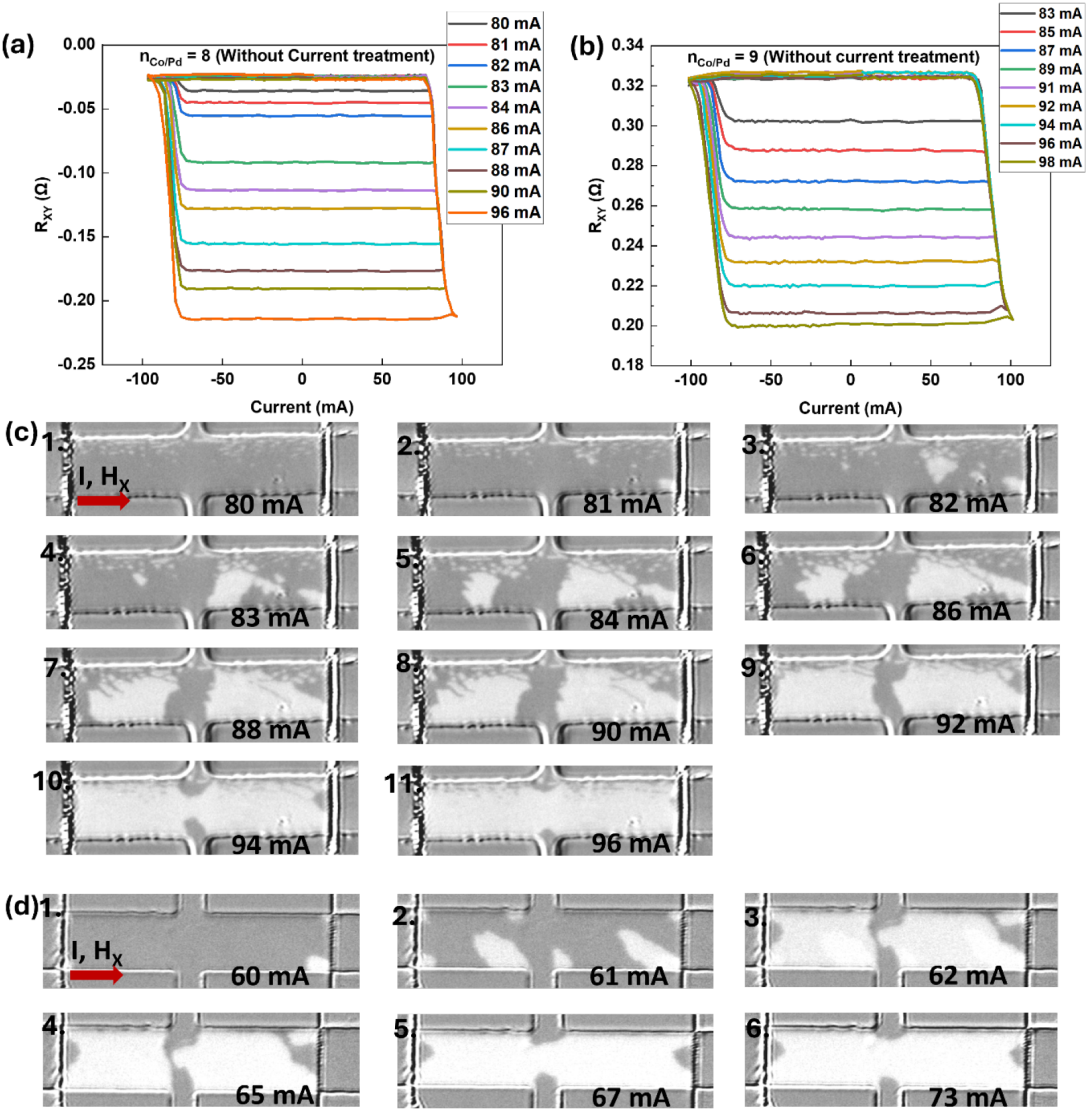
Analog-only SOT switching in as-fabricated PtMn/(Co/Pd)_n=8–9_ devices. (a) Multilevel (analog) SOT switching for n_Co/Pd_ = 8, (b) Multilevel (analog) SOT switching for n_Co/Pd_ = 9, (c) In-situ Kerr microscopy for n_Co/Pd_ = 8 showing nucleation-dominated/suppressed propagation reversal with limited domain-wall propagation, and (d) Kerr imaging of the n_Co/Pd_ = 5 device depicting SOT switching by nucleation and propagation. Each pulse is 300 μs long, and H_X_ = 40 Oe for both SOT switching and in situ Kerr imaging.

The dual SOT modes observed in the devices with for n_Co/Pd_ = 2–7 originate from current-induced twin formation and defect-mediated pinning due to the large current treatment.^34^ By increasing the number of Co/Pd repeats alone, we demonstrate that we can stabilize the analog SOT regime in the as-fabricated devices. The increased repeats with thicker stacks likely accumulate a higher twin defect density, which enhances pinning and thereby suppresses domain-wall propagation, favoring nucleation-driven reversal from the outset. Thus, by controlling the multilayer architecture rather than relying on postprocessing, we can deterministically select analog behavior, providing a straightforward materials-level handle for designing robust spintronic synapses. This n_Co/Pd_ dependent mode selection also exists at shorter pulses, as shown in Supporting Information S3 using 10 μs long pulse SOT measurements of representative devices n_Co/Pd_ = 3, 5 (binary SOT in as-deposited devices) and n_Co/Pd_ = 8 (analog SOT in as-deposited devices).

### 3.4 Hybrid Structural–Electrical Tuning of High-n Multilayers

To further examine how current treatment influences the analog SOT behavior in already analog-only as-fabricated devices (n_Co/Pd_ = 8 and 9), we applied progressively higher current amplitudes until a measurable increase in the channel resistance (R_xx_) was detected, indicating structural modification. Figure 4a presents the evolution of R_xx_ with increasing current for both n_Co/Pd_ = 8 and 9. Figure 4b and 4c show the SOT switching curves after this high-current treatment for n_Co/Pd_ = 8 and n_Co/Pd_ = 9 respectively. Compared to the pristine analog behavior shown previously in Figure 3a and 3b, the post-treatment devices exhibit a much more gradual resistance evolution and a significantly larger number of intermediate states, indicating an enhanced analog SOT response.

**Figure 4:**
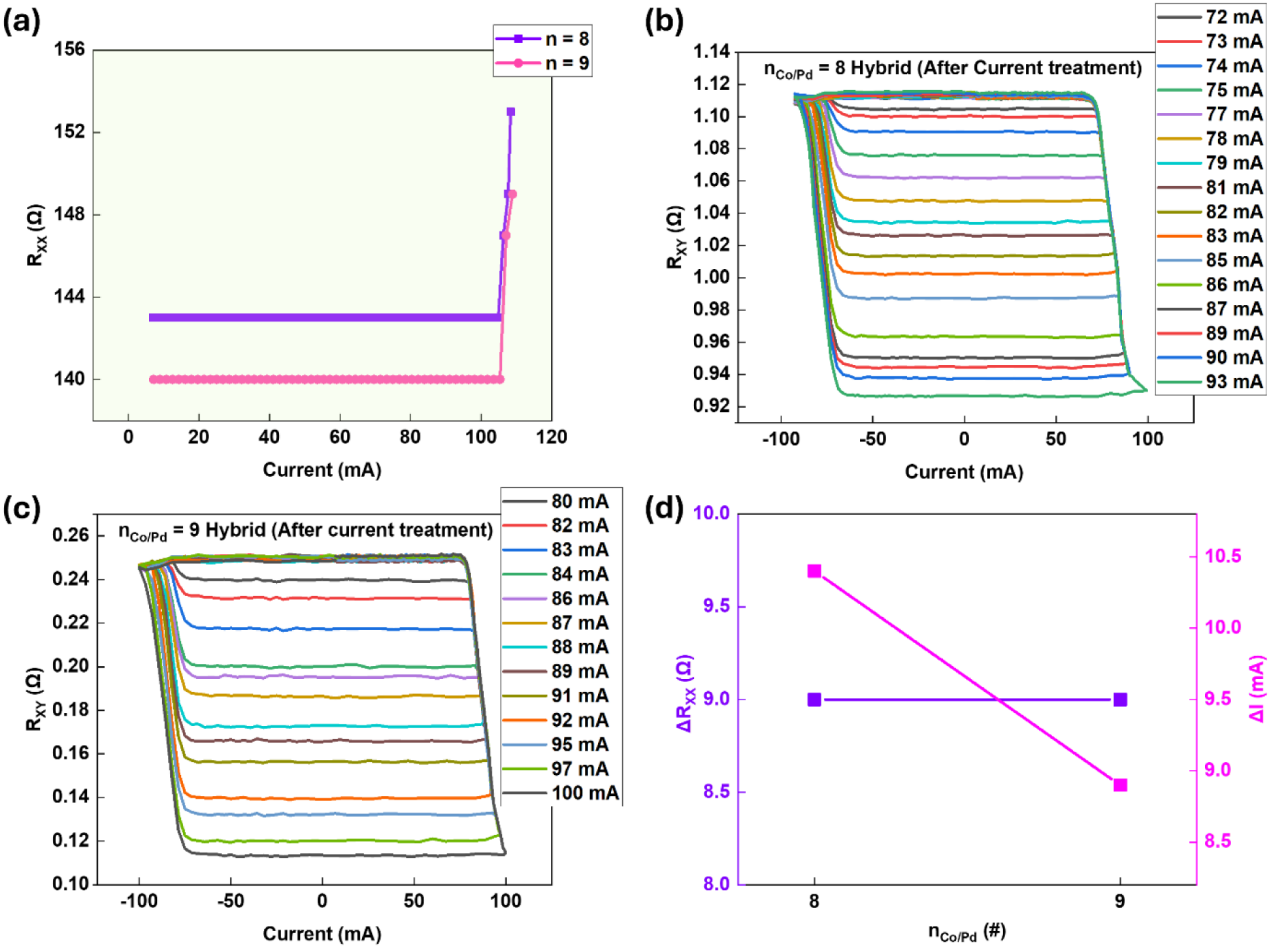
Hybrid control of analog SOT in PtMn/(Co/Pd)_n=8,9_. (a) Evolution of longitudinal resistance R_xx_ with increasing current amplitude for each n_Co/Pd_ = 8, and n_Co/Pd_ = 9. (b, c) Enhanced analog SOT switching after high-current treatment for n_Co/Pd_ = 8 and n_Co/Pd_ = 9, respectively, showing more gradual resistance change and an increased number of intermediate states compared with pristine analog devices. (d) Summary of resistance change (ΔR_XX_) and the additional current (ΔI) required to achieve the hybrid regime for samples with n_Co/Pd_ = 8 and n_Co/Pd_ = 9. All measurements use 300 μs pulses at H_x_ = 40 Oe.

We term this state the hybrid mode, achieved by combining structural design (via multilayer repeat number) with electrical conditioning. This dual control provides a powerful means to finely tune the analog switching characteristics without compromising device stability. Unlike the analog behavior induced by high-current or annealing treatment in thinner stacks n_Co/Pd_ ≤ 7, where analog behavior arises after a structural change, the hybrid mode in thicker stacks leverages their inherently high pinning density further to refine device analog behavior through controlled current stimulation. Figure 4d summarizes the change in R_xx_ (ΔR_XX_) and the additional current (ΔI) required to achieve the hybrid regime for each n_Co/Pd_ = 8 and 9. For n_Co/Pd_ ≥ 8, the films are intrinsically analog in the as-deposited state; mild current conditioning (ΔI) further enhances twin density (from 0.52 to 0.72 nm^–1^ for n_Co/Pd_ = 8) (as discussed later in Figure 5), making switching even more gradual and thus enhancing the number of states. In contrast, excessive conditioning eventually overpins the walls and narrows the switching window. For n_Co/Pd_ = 8 and n_Co/Pd_ = 9, ΔR_XX_ remains about ∼9 Ω after current conditioning because the Co/Pd multilayers are already highly twinned, leaving limited headroom for additional defect-induced resistivity changes. Parallel shunting through the Pt/PtMn/Ta layers further constrains the device-level resistance increase. Together, the monotonic decrease in ΔI with n_Co/Pd_ and initial monotonic rise of ΔR_XX_, and then nearly constant at high n_Co/Pd_ confirm that enhanced twin density and interface disorder progressively lower the energetic threshold for nucleation-dominated analog switching, establishing a clear structural–electrical correlation governing the binary-to-analog transition.

**Figure 5:**
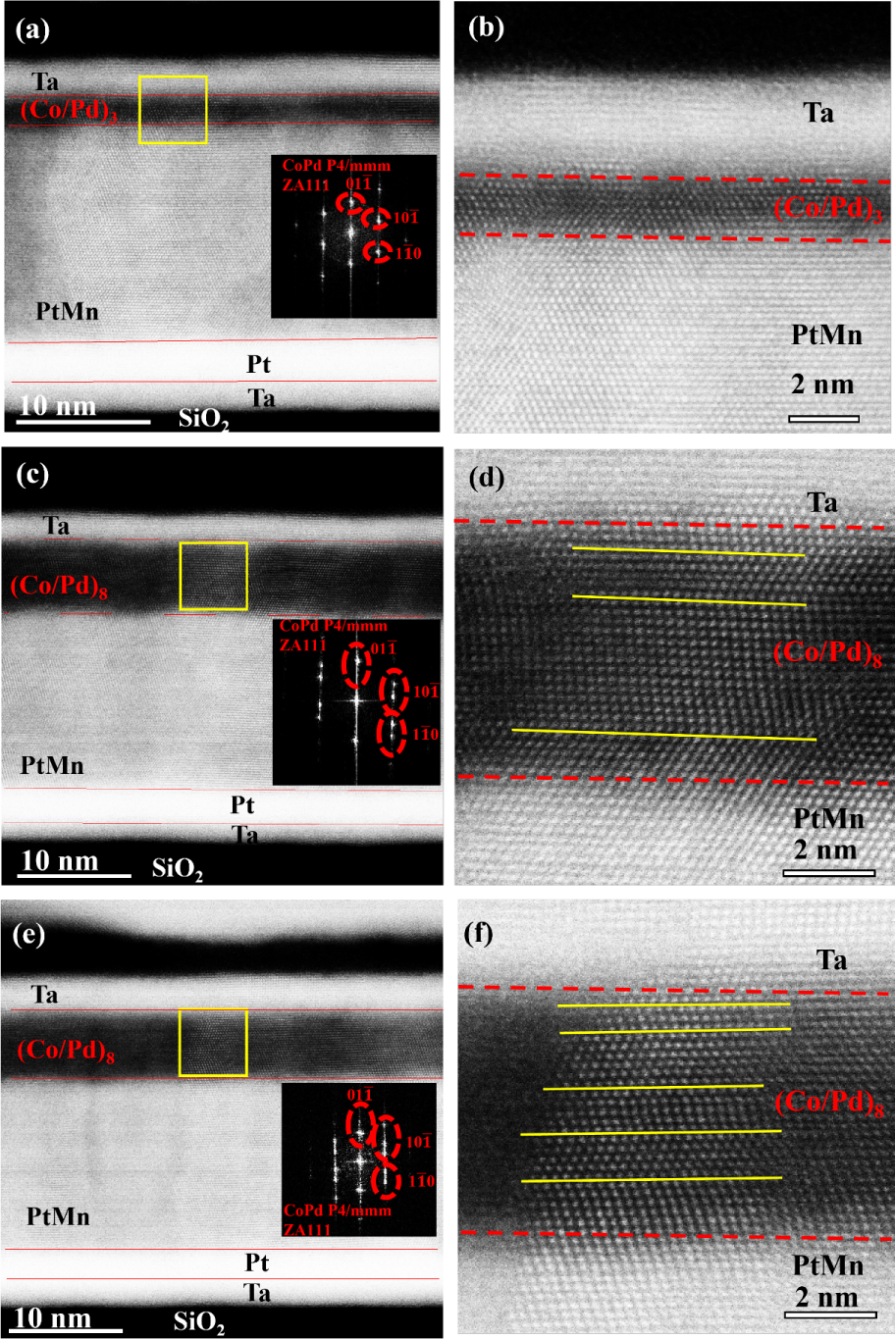
Cross-sectional HRTEM images revealing twin evolution vs repeat number and current treatment. (a, b) n_Co/Pd_ = 3 (as-deposited): whole stack and high-magnification images to show CoPd regions with corresponding FFTs. Co/Pd multilayers are smooth and uniform; no visible twins or FFT streaking—consistent with low pinning and binary SOT. (c, d) n_Co/Pd_ = 8 (as-deposited): increased twin boundaries within CoPd (and locally in PtMn); measured CoPd twin density ∼0.52 nm^–1^. Analog-only SOT is observed in this state. (e, f) n_Co/Pd_ =8 after high-current treatment (hybrid): CoPd has the highest twin density ∼0.72 nm^–1^ with the most pronounced FFT streaking, indicating enhanced lattice distortion/interface shear; correlates with the most gradual multilevel SOT.

Furthermore, to test the stability and reproducibility of the hybrid-treated devices and to directly evaluate post-treatment robustness under severe operational conditions, we performed an accelerated cycling test on hybrid devices using 100 write pulses (pulse width: 300 μs) with an amplitude of approximately 95 mA, i.e., the current required to complete the switching. After the test, the switching portion decreased only slightly (≈3%) after 100 pulses, without any change in current channel resistance (R_XX_), indicating that the hybrid state is robust against repetitive high-amplitude stressing and does not exhibit rapid degradation. The stability of the multilevel states of these hybrid devices under 100 and 200 writing-pulse thermal stress tests is discussed in detail in Supporting Information S4.

### 3.5 Microstructural Origin of the Mode Crossover

To reveal the microstructural origin of the observed SOT-mode transition upon increasing n_Co/Pd_ and explain why the hybrid one provides the best analog behavior, we performed cross-sectional HRTEM on three representative stacks: n_Co/Pd_ = 3 (as-deposited), n_Co/Pd_ = 8 (as-deposited), and n_Co/Pd_ = 8 after high-current treatment (hybrid). Figure 5 presents the corresponding low- and high-magnification images as well as FFT patterns. All samples maintain a well-textured (111)-oriented PtMn antiferromagnetic layer and columnar multilayer stacking with sharp interfaces. From the Fast Fourier Transforms (FFTs) and lattice imaging, we also verify that the Co and Pd multilayers form an L1_0_-ordered Co_50_Pd_50_ alloy with a tetragonal crystal lattice (*P*4/*mmm*) in all samples. It has been previously shown that Co and Pd are more likely to form alloys rather than remain as superlattices.^38^

Besides TEM analysis of n_Co/Pd_ = 8 (without any current treatment), we also performed TEM n_Co/Pd_ = 8 (Hybrid) analysis to see if the improved analog behavior in the hybrid case is due to enhancement of twin defects after current treatment as previous study had established that current-induced twin formation within Co/Pd and PtMn layers is the microscopic origin of the binary-to-analog SOT transition.^34^ There, the transition occurred solely via electrical conditioning without altering stack layer or device geometry contingent upon current treatment, directly linking twin-induced domain pinning to nucleation-dominated analog switching. Here, we extend that understanding to test whether a purely structural route, i.e., varying the Co/Pd repeat number n_Co/Pd_ and then further applying the current treatment, can independently modulate twin density and thus control the SOT mode.

For n_Co/Pd_ = 3 (Figure 5a and 5b), the CoPd layer appears smooth and uniform, and no evident twin contrast or FFT streaking is observed, consistent with low pinning and fast domain-wall propagation that yields binary SOT in the as-deposited state. In contrast, the as-depositedn_Co/Pd_ = 8 film (Figure 5c and 5d) exhibits a clear increase in twin boundaries within the CoPd region, with a measured twin density ∼0.52 nm^–1^, confirming that microstructural changes with repeat number increase. This enhanced twinning supplies additional pinning sites and explains the analog-only SOT observed without current or thermal treatment in n_Co/Pd_ = 8/9. After controlled high-current conditioning, the n_Co/Pd_ = 8 (hybrid) sample (Figure 5e and 5f) shows the highest twin density ∼0.72 nm^–1^ and the most pronounced FFT streaking, indicating increased lattice distortion and interface shear. Note that the yellow line marks are representative of twin planes in Figure 5d and 5f. This correlates directly with the most gradual multilevel SOT response (Figure 4), which exhibits better analog behavior, demonstrating that twin-induced pinning suppresses long-range propagation and promotes nucleation-dominated reversal. The progression n_Co/Pd_ = 3 → 8 → 8 (hybrid), therefore, maps quantitatively onto increasing twin density and analog behavior, extending the previous findings^34^ to include purely structural and hybrid pathways for achieving analog SOT. Besides the HRTEM analysis, the roughness measured by AFM reveals a quite low value (∼100 pm) in all samples. The results are shown in Supporting Information S5. These roughness values indicate that the switching-mode evolution contingent upon increasing n_Co/Pd_ is primarily governed by defect (nucleation-site) density (twin density) increase with n_Co/Pd_ rather than surface roughness.

All samples show a (111)-textured PtMn layer and columnar multilayer stacking; FFTs confirm L10-ordered Co_50_Pd_50_ (*P*4/*mmm*) in the Co/Pd region. The yellow line marks represent twin planes. Scale bars: 10 nm (left column) and 2 nm (right column).

### 3.6 Integration with Field-Free SOT Switching

In this work, we did not perform any annealing, which is typically required in PtMn-based stacks to establish a robust in-plane exchange bias that can serve as an internal symmetry-breaking field for field-free SOT switching.^2^ Our deliberate choice was to demonstrate that deterministic control of SOT switching modes (binary-like vs analog-like) can be achieved in the as-deposited state through a materials-level design parameter, namely the Co/Pd repeat number n_Co/Pd_. This enables cointegration of binary-mode devices and analog synapses within a single deposition/fabrication flow, while still leaving room for optional “hybrid tuning” steps (e.g., annealing/field-cooling and/or current treatment) when field-free operation is desired.

Feasible routes toward field-free switching that are compatible with the PtMn/(Co/Pd)­n platform in this work, includes: (i) engineering an in-plane exchange bias in PtMn-based stacks (e.g., via field annealing or field-cooling protocols),^2,25^ (ii) introducing structural asymmetry (e.g., wedge structures, asymmetric oxidation/capping, or device-level geometric asymmetry),^26^ and (iii) DMI engineering and tilted-anisotropy approaches to provide intrinsic symmetry breaking at zero external field.^39–41^

Consistent with our TEM-identified increase in twin density with n_Co/Pd_, prior field-driven studies on Co/Pd multilayers have shown that increasing n_Co/Pd_ systematically roughens the domain wall (DW) and alters the reversal pathway. Sharma et al. reported a progression from smooth walls to highly rough, dendritic-like DWs as n_Co/Pd_ increases from 1 to 9, with propagation becoming patchy at large n_Co/Pd_ due to increased DW roughness.^42^ Choe et al. showed that raising the repeat number suppresses wall-motion speed while leaving the nucleation rate comparatively higher, driving a wall-motion to nucleation-dominated crossover.^43,44^ In parallel, proximity-induced polarization of thin Pd by adjacent Co increases M_S_ as repeats are added, elevating the dipolar energy $K_{d}=\mu_{0}M_{s}^{2}/2$ and lowering the quality factor Q = K_eff_/K_d_; where K_eff_ = M_S_H_K_/2. This reduced Q promotes DW deformation and pinning, further promoting nucleation-dominated reversal at high n_Co/Pd_.^42–44^ Taken together, these reports support our findings: as n_Co/Pd_ grows, enhanced pinning (here, via increased twin density) and DW roughening suppress long-range propagation, yielding the analog-only SOT observed for n_Co/Pd_ ≥ 8, while lower n_Co/Pd_ permits nucleation and propagation, leading to a binary behavior in the as-deposited state. The large current treatment for devices with a lower n_Co/Pd_ enhances the twin formation, resulting in analog behavior.

### 3.7 Synaptic Plasticity and Weight Update Behavior

We benchmarked synaptic plasticity for analog devices realized by each route, i.e., current treatment, material selection, and hybrid combination, by comparing cycle-to-cycle reproducibility, asymmetry, and nonlinearity. Long-term potentiation/depression (LTP/LTD) is realized while reading the anomalous Hall voltage V_AHE_ as the synaptic weight (Figure 6a–6c). Starting from −M_z_ and with an in-plane field H_x_ = 40 Oe, we applied 300 μs long pulses separated by 1 s. For LTP, a train of negative pulses with gradually increasing amplitude drove a monotonic rise in V_AHE_; for LTD, a train of positive pulses with increasing amplitudes produced a monotonic decrease. We repeated these write–erase sequences over multiple cycles and compared three representative cases: Figure 6a is n_Co/Pd_ = 5 after the current treatment (dual-mode device operated in analog), Figure 6b is n_Co/Pd_ = 8 as-deposited (intrinsically analog), and Figure 6c is n_Co/Pd_ = 8 after additional current conditioning (hybrid). All three show multilevel updates, with the hybrid sample exhibiting the smoothest evolution and the largest number of intermediate states and thus the best synaptic plasticity.

**Figure 6:**
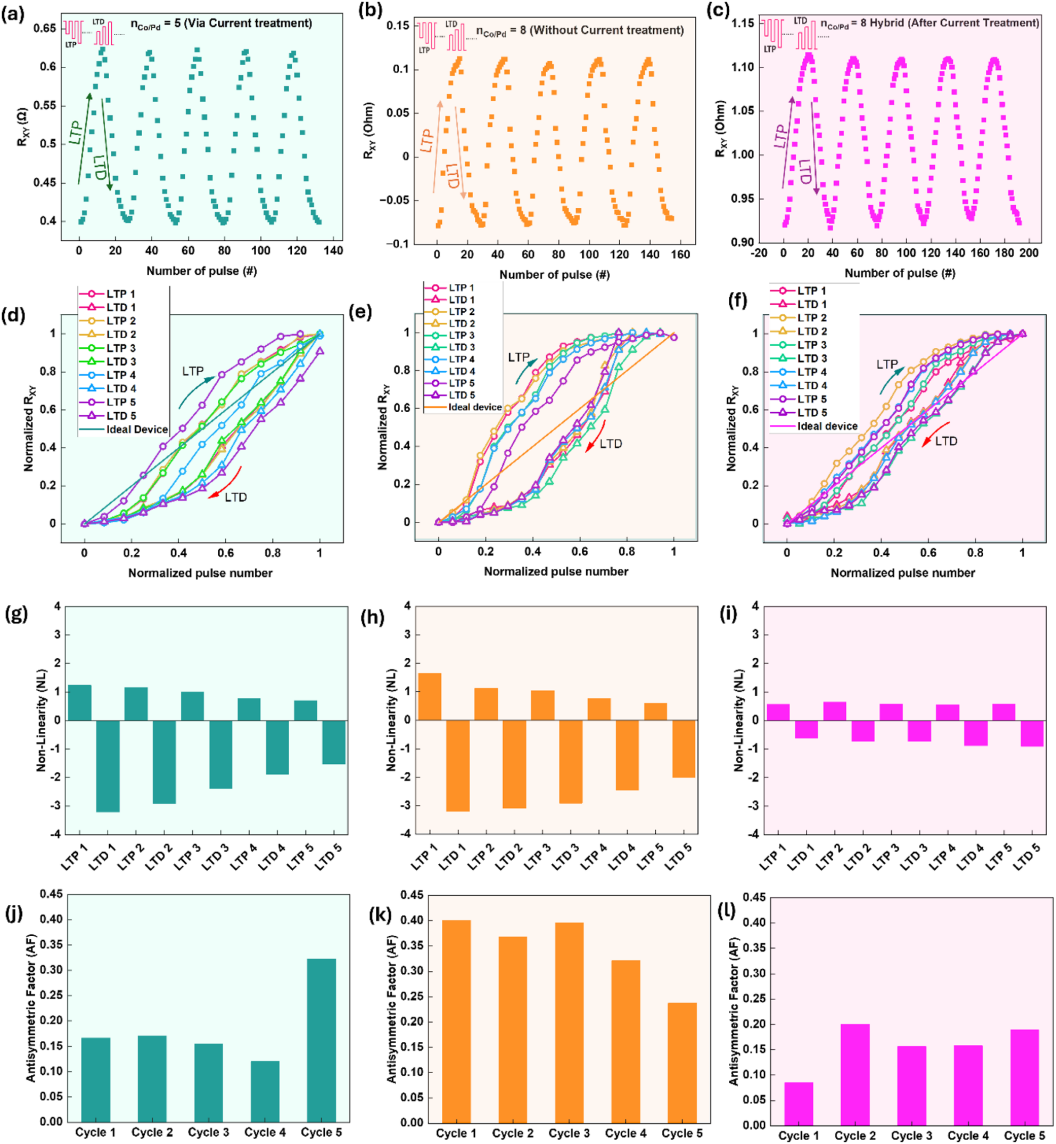
Synaptic plasticity performance for three analog SOT devices. Repeated LTP/LTD curve depicting the repeatability of the stability, linearity, and symmetry of synaptic weights update for (a) n_Co/Pd_ = 5 with a large current treatment, (b) n_Co/Pd_ = 8 without current treatment, (c) n_Co/Pd_ = 8 (Hybrid, after current treatment). Normalized LTP and LTD curves over various cycles for (d) n_Co/Pd_ = 5 with a large current treatment, (e) n_Co/Pd_ = 8 without current treatment, and (f) n_Co/Pd_ = 8 (Hybrid, after current treatment). Nonlinearity factor (NL) of the LTP/LTD for various measurement cycles for (g) n_Co/Pd_ = 5 with a large current treatment, (h) n_Co/Pd_ = 8 without current treatment, (i) n_Co/Pd_ = 8 (Hybrid, after current treatment). Antisymmetric factor (AF) for various cycles of LTP/LTD measurements for (j) n_Co/Pd_ = 5 with a large current treatment, (k) n_Co/Pd_ = 8 without current treatment, and (l) n_Co/Pd_ = 8 (Hybrid, after current treatment). LTP/LTD were performed under H_x_ = 40 Oe with a 300 μs-long train of pulses with increasing amplitudes with 1 s spacing between them.

Linearity and symmetry were also quantified over repeated LTP/LTD cycles (Figure 6d–6l) for the above three cases. Normalized LTP and LTD curves over various cycles for n_Co/Pd_ = 5 with a large current treatment, n_Co/Pd_ = 8 without current treatment, and n_Co/Pd_ = 8 (Hybrid, after current treatment) are shown in Figure 6d–6f, respectively. We plotted normalized V_AHE_ versus normalized pulse number and fitted the trajectories using the method given by Chen, Pai-Yu, et al, from which NL_LTP_ and NL_LTD_ were calculated.^45^ The following equations are used to calculate the nonlinearity factor (NL_LTP_ and NL_LTD_).

1 $$G_{pot}=B\left(1-e^{\left(-\frac{P}{A}\right)}\right)+G_{min}$$

2 $$G_{dep}=-B\left(1-e^{(\frac{P-P_{\max}}{A})}\right)+G_{max}$$

3 $$B=-\left(G_{max}-G_{min}\right)/\left(1-e^{(\frac{-P_{\max}}{A})}\right)$$

Here, *G*_*pot*_ and *G_dep_* are the V_AHE_ values for potentiation and depression, respectively. *P* is the number of pulses, *P_max_* is the maximum number of pulses, *G*_*max*_ and *G*_*min*_ are the maximum conductance and the minimum conductance, respectively. Parameter *A* determines the nonlinear behavior of the synaptic weight update. *B* is a function of *A* that fits the functions within the range of *G_max_*, *G_min_* and *P_max_*. According to the lookup table provided by Chen, Pai-Yu et al., the NL_LTP_ and NL_LTD_ are evaluated based on the value of *A*.^45^

The symmetry or the antisymmetric factor (AF) of the potentiation and depression curves is determined by calculating the root-mean-square deviation (RMSD) between the LTP and LTD curves using the formulas:

4 $$\mathrm{AF}=\sqrt{\frac{1}{N}\underset{i=1}{\sum }\left(f(x\right)-g\left(x\right){)}^{2}}$$

Here, *f*(*x*) and *g*(*x*) refer to the potentiation and depression plots, respectively. The antisymmetric factor (AF) values indicate asymmetry between the LTP and LTD curves.^45^

Using the models and equations described earlier, we calculated the Nonlinearity of LTP (NL_LTP_), Nonlinearity of LTD (NL_LTD_), and AF for five LTP/LTD cycles across three device types: current-treated devices (n_Co/Pd_ = 5), material-selected devices (n_Co/Pd_ = 8), without current treatment, and hybrid devices (n_Co/Pd_ = 8), after current treatment. The NL_LTP_ and NL_LTD_ values for each case are plotted in Figure 6g–6i. For n_Co/Pd_ = 5, the average NL_LTP_ is 0.98 and the average NL_LTD_ is −2.382. For the n_Co/Pd_ = 8 device without current treatment (material-selected), the average NL_LTP_ is 1.032 and the average NL_LTD_ is −2.728. In the hybrid case, with the current treatment (n_Co/Pd_ = 8), the average NL_LTP_ is 0.59 and the average NL_LTD_ is −0.79. The observed difference between Nonlinearity of LTP (NL_LTP_) and Nonlinearity of LTD (NL_LTD_) in the current treated n_Co/Pd_ = 5, and as deposited n_Co/Pd_ = 8 is discussed in detail in Supporting Information S6.

The AF values for the three cases are plotted in Figure 6j–6l. The average AF over five cycles for n_Co/Pd_ = 5 is 0.1867, for n_Co/Pd_ = 8 without current treatment is 0.3445, and for the hybrid case with current treatment (n_Co/Pd_ = 8) is 0.1577.

These results clearly demonstrate that the hybrid mode (a combination of stack design and current treatment) significantly improves synaptic device performance, as evidenced by the lower NL and AF values. The hybrid mode outperforms both the current-treated device and the material-selected device, confirming its superior synaptic plasticity with more stable, linear, and symmetric potentiation and depression. The superior performance of the hybrid mode may originate from its optimized microstructure and the resulting SOT dynamics. As revealed by HRTEM images (Figure 5), current treatment of the high-n_Co/Pd_ stack further increases twin density (from 0.52 to 0.72 nm^–1^ for n_Co/Pd_ = 8), introducing additional planar defects and interface shear within the CoPd region. These extended twin boundaries serve as uniform pinning sites, suppressing long-range domain-wall propagation and promoting nucleation-dominated reversal. Such localized, distributed switching leads to a dense hierarchy of intermediate magnetic states with reduced stochasticity, which manifests as smoother, more symmetric potentiation and depression curves (lower NL and AF in Figure 6).

In contrast, purely current-treated (low-n_Co/Pd_) devices generate spatially nonuniform twin distributions, causing irregular pinning and higher variation in state spacing, while as-deposited high-n_Co/Pd_ devices exhibit limited tunability of their preexisting defect structure. The hybrid configuration therefore achieves an ideal balance between structural design and controlled defect modulation, providing sufficient pinning to stabilize analog states yet maintaining enough mobility for reproducible, incremental weight updates. This synergy between microstructural engineering and electrical conditioning directly links the observed SOT multilevel linearity and symmetry to atomic-scale defect control, explaining the superior synaptic precision.

### 3.8 Neuromorphic Computing Performance

To identify the most suitable device for optimal neuromorphic potential among the PtMn/(Co/Pd)­n/Ta devices, we performed artificial neural network (ANN) simulations to classify MNIST digits/letters using a Multilayer Perceptron (MLP) architecture.^45–48^ This evaluation enabled us to assess the impact of various device configurations (current-treated, material-selected, and hybrid modes) on the overall performance of neuromorphic tasks, providing insights into which device offers the optimal balance of synaptic plasticity, stability, and classification accuracy. To evaluate the neuromorphic performance of the PtMn/(Co/Pd)­n devices, we incorporated their synaptic behaviors into a multilayer perceptron (MLP) neural network for the MNIST digit classification task. As illustrated in (Figure 7a), the MLP consists of three layers: 784 input neurons corresponding to the 28 × 28-pixel MNIST images, a hidden layer with 100 neurons, and an output layer with 10 neurons representing the digit classes (0–9).^45–48^ Synaptic weights in the network are derived from experimentally measured V_AHE_ values of the PtMn/(Co/Pd)­n/Ta devices.

**Figure 7:**
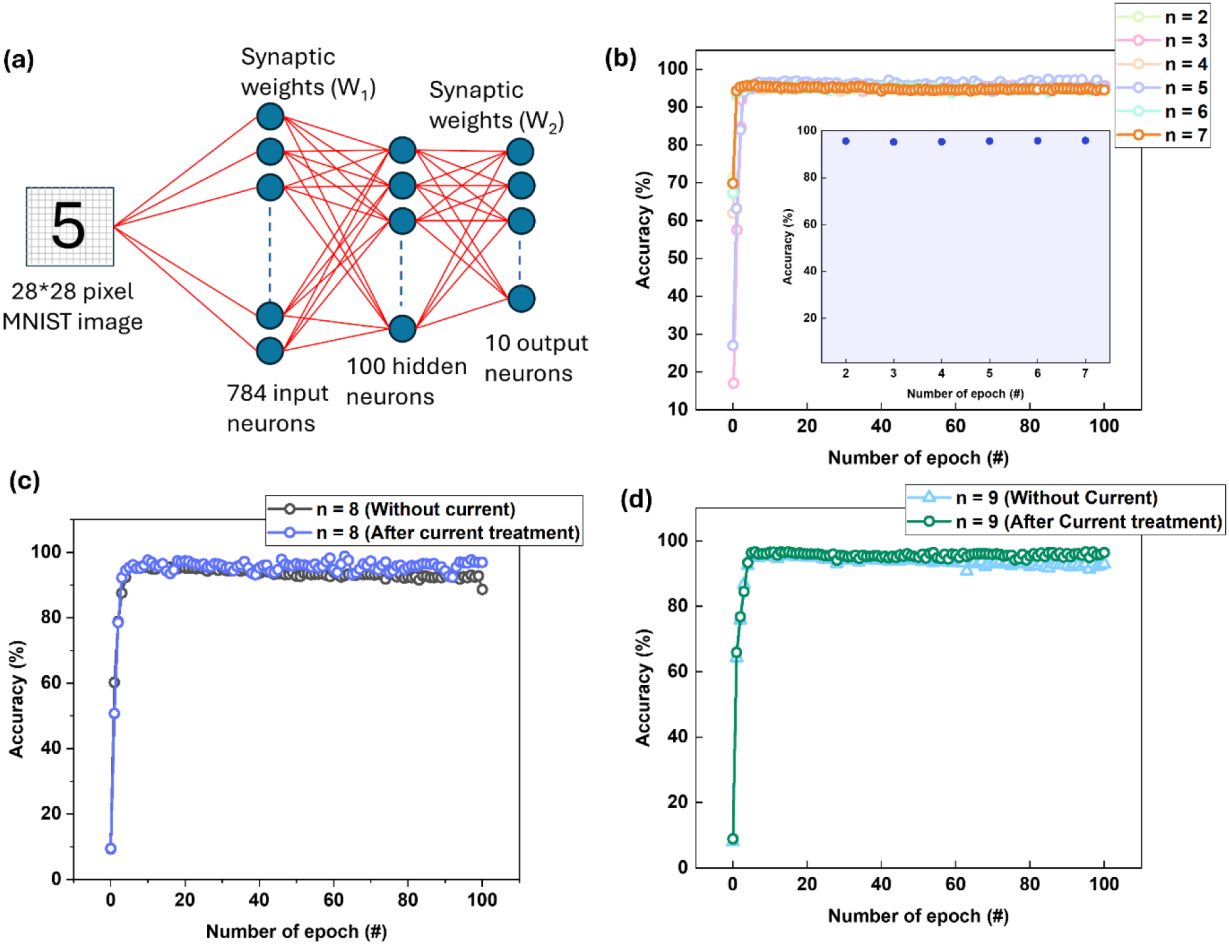
Demonstration of neuromorphic device performance. (a) Schematic of the Multilayer Perceptron (MLP) used in the artificial neural network simulation for MNIST digit recognition, (b) The recognition accuracy for current-treated dual mode samples (n_Co/Pd_ = 2–7), with all samples achieving accuracy in the range of 95–96%, (c) The recognition accuracy for n_Co/Pd_ = 8 devices (without current-treated and after current treated (Hybrid)), with hybrid samples achieving higher accuracy of over 97%, and (d) The recognition accuracy for n_Co/Pd_ = 9 devices (without current-treated and after current treated (hybrid)), with hybrid samples achieving higher accuracy of over 97%.

The weights in the software were first normalized to a range of [−1, 1], and then a linear transfer function was applied to map these values to the physical states of the device, ensuring a one-to-one correspondence between the computational weights and accessible experimental states. This normalization process improves the convergence and stability of the training process.

To emulate real-world hardware imperfections, we injected additive noise into the V_AHE_ values and simulated stuck-at-minimum faults, representing nonideal behavior commonly seen in physical devices. These perturbed V_AHE_ values were then mapped back to the weights used in the neural network, allowing the MLP to operate with realistic device variability while maintaining functional accuracy. Figure 7b shows the recognition accuracy for current-treated dual-mode samples (n_Co/Pd_ = 2–7), with all samples achieving an accuracy range of 95–96%, consistent with the results previously obtained for n_Co/Pd_ = 4.^34^ This demonstrates the flexibility of selecting the best dual SOT device that offers optimal performance and thermal stability.

Figure 7c and 7d present the recognition accuracy for the samples of n_Co/Pd_ = 8 (with and without current treatment) and n_Co/Pd_ = 9 (with and without current treatment), respectively. For the n_Co/Pd_ = 8 and n_Co/Pd_ = 9 devices without current treatment, the recognition accuracy remained similar to that of the current-treated dual devices, around 96%. However, for the hybrid devices (*n*_Co/Pd_ = 8 and *n*_Co/Pd_ = 9 after current treatment), the recognition accuracy increased slightly from ∼96% to over 97%, indicating a modest improvement enabled by the hybrid approach. This improved accuracy can be attributed to enhanced intermediate states and better linearity and symmetry responses of LTP/LTD synaptic weight updates. This underscores the superior potential of combining material selection with current conditioning to optimize synaptic plasticity and overall device performance for neuromorphic computing applications.

## 4 Conclusion

We demonstrate that the Co/Pd repeat number in PtMn/(Co/Pd)_n_ multilayers provides a direct and deterministic route to select the SOT switching mode and synaptic functionality. Increasing n_Co/Pd_ modifies the multilayer microstructure in a systematic manner, including greater interface modulation and the formation of correlated planar defects, such as twins. These collective structural changes alter the switching dynamics between nucleation and domain-wall propagation during magnetization reversal. For the as-deposited sample with n_Co/Pd_ ≤ 7, domain-wall propagation remains efficient, resulting in binary switching that can transition to analog behavior only after additional defect formation through electrical conditioning. In contrast, for n_Co/Pd_ ≥ 8, the increased structural modulation in the as-deposited state suppresses long-range propagation and stabilizes nucleation-dominated analog switching without any post-treatment. Furthermore, combining a high repeat number with controlled current conditioning produces a refined structural landscape characterized by a more uniform and spatially distributed pinning environment. This hybrid state enables smooth, incremental magnetization updates, expands the number of accessible intermediate magnetic states, and improves both the linearity and symmetry of long-term potentiation and depression. As a result, hybrid devices with n_Co/Pd_ ≥ 8 achieve recognition accuracy in the MNIST classification task exceeding 97%. These findings demonstrate that the switching pathway of SOT devices can be tuned to enhance synaptic responses by tailoring the multilayer stacking architecture and applying electrical current conditioning. This work provides a scalable strategy for constructing thermally stable, energy-efficient analog spintronic synapses suitable for high-performance neuromorphic hardware.

## Supplementary Material



## Data Availability

The data that support the findings of this study are available from the corresponding author upon reasonable request.

## Notes

Loop-shift measurements for DMI field and damping-like SOT efficiency extraction; pulse-width dependence of SOT switching and mode selection; accelerated cycling/thermal-stress endurance and reproducibility of hybrid/dual-mode devices; AFM surface-roughness analysis versus n_Co/Pd_; additional analysis of LTP/LTD nonlinearity metrics; supplementary figures and tables (PDF)
