# Supplementary material for: Stack-Engineered Mode Selection in PtMn/(Co/Pd)n Multilayers Enables Deterministic Analog Spin–Orbit Torque Synapses
Source: ACS Appl Mater Interfaces. 2026 Mar 11;18(11):16662–74. doi: 10.1021/acsami.5c22532 (PMC13022814; doi:10.1021/acsami.5c22532)
Supplement: Supplementary file 1 [file am5c22532_si_001.pdf]

Supporting Information

Stack-Engineered Mode Selection in PtMn/(Co/Pd)□ Multilayers Enables Deterministic Analog Spin–Orbit Torque Synapses

*Abhijeet Ranjan[1], Tamkeen Farooq[2], Chong-Chi Chi[3], Chao-Chin Wang[4], Yu-Lon Lin[5], Rudis Ismael Salinas Padilla[1], Ying-Hung Li[1], Yuan-Chieh Tseng[5,6], Chih-Hao Lee[4], Ming-Yen Lu[1,3,7], Rahul Mishra[2] and Chih-Huang Lai[1,7a]**

Affiliations:

1. Department of Materials Science and Engineering, National Tsing Hua University, Hsinchu 30013, Taiwan.
2. Centre for Applied Research in Electronics, Indian Institute of Technology Delhi, New Delhi 110016, India.
3. Instrumentation Center, National Tsing Hua University, Hsinchu 300, Taiwan.
4. Department of Engineering and System Science, National Tsing Hua University, Hsinchu 30013, Taiwan.
5. Department of Materials Science & Engineering, National Yang-Ming Chiao-Tung University, Hsinchu 30010, Taiwan

6. Institute of Space Systems Engineering, National Yang-Ming Chiao-Tung University, Hsinchu 30010, Taiwan.

7. College of Semiconductor Research, National Tsing Hua University, Hsinchu 30013, Taiwan.

[a*]Author to whom correspondence should be addressed: chlai@mx.nthu.edu.tw.

KEYWORDS: Spin-Orbit Torque, Binary switching, Multi-level Switching, Neuromorphic Computing, Artificial Synapses, Artificial Neural Networks.

Supplementary Information S1: Loop shift measurements for DMI field and SOT efficiency calculations.

Loop shift method developed by Pai et al. can be used to quantify the damping-like effective field H_z^{eff} and the DMI field of the HM/FM systems[1-2]. It is obtained from the shift of the anomalous Hall hysteresis loop under DC current at a fixed in-plane bias field H_x, and the DL efficiency is computed from the loop-shift slope H_z^{eff}/I. The DL torque efficiency is written as

$$\xi_{\mathrm{DL}} = \left(\frac{2}{\pi}\right)\left(\frac{2e}{\hbar}\right)u_0 M_s t_{FM} w t_{HM}(1+s)\left(\frac{H_z^{Eff}}{I}\right) \quad \text{(S1)}$$

where $s = \frac{t_{FM}\rho_{HM}}{t_{HM}\rho_{FM}}$. Here, e is the elementary charge, ℏ is the reduced Planck's constant, u_0 is the permeability of vacuum, M_S is the saturation magnetization of the ferromagnet, t_{FM} is the thickness of the ferromagnet, ρ_{HM} is the resistivity of the heavy metal, and ρ_{FM} is the resistivity of the ferromagnet, w is the width of current channel, t_{HM} is the thickness of the HM, s denotes the amount

of current going into the HM, $\frac{H_z^{Eff}}{I}$ is the saturated effective field per unit current calculated from loop shift measurement[1,2].The setup and the loop shift measurement results for one of the representative devices, $n_{Co/Pd}$ = 5, are shown below in the supplementary Figure S1. Loop shift measurement setup is shown in Figure S1 (a). The shift of field AHE loops for $\pm$31 mA DC current under in plane magnetic field H_X = 150 Oe is shown below in Figure S1 (b). H_z^{Eff} vs I under H_X = 150 Oe is shown below in Figure S1 (c), and $\frac{H_z^{Eff}}{I}$ Vs H_X is plotted in Figure S1 (d). From Figure S1 (d) we calculated H_{DMI} and saturated $\frac{H_z^{Eff}}{I}$ to be 50 Oe, and 1.4 Oe/mA, respectively. The low H_{DMI} scales very well with low H_X requirements during SOT measurements. And using saturated $\frac{H_z^{Eff}}{I}$ = 1.4 Oe/mA we can calculate the damping like SOT efficiency to be: 0.235, Θ_{SH} can be calculated from ξ_{DL} using the equation $\xi_{DL} = T_{int}\ \Theta_{SH}$, where T_{int} is the interfacial spin transparency. Note that it includes the contributions from both PtMn and the Top Ta[3,4].

The saturated value of H^Z_{eff}/ I gives the DL efficiency, while the H_X at which H^Z_{eff}/ I is saturated gives the H_{DMI} field.

These values are comparable order of magnitude to the reported values for PtMn[3,6], the increased magnitude can be explained using the fact that a higher effective (combined) torque efficiency is plausible when the ferromagnet is sandwiched between two spin–Hall-active metals (PtMn and Ta in this case), because torques from the top and bottom layers can add constructively. For example, Woo et al. reported that in Pt/Co/Ta stacks the effective fields and switching efficiency increase with Ta overlayer thickness, yielding an effective spin Hall angle up to ~0.34 , which they attribute to the combined action of the two spin–Hall layers and interfacial effects (e.g., intermixing/graded interfaces)[5].

We can clearly see that efficiency depends not only on HM but also on FM owing to the interface-related factors[3,6]. The comparison is shown below in Table S1.

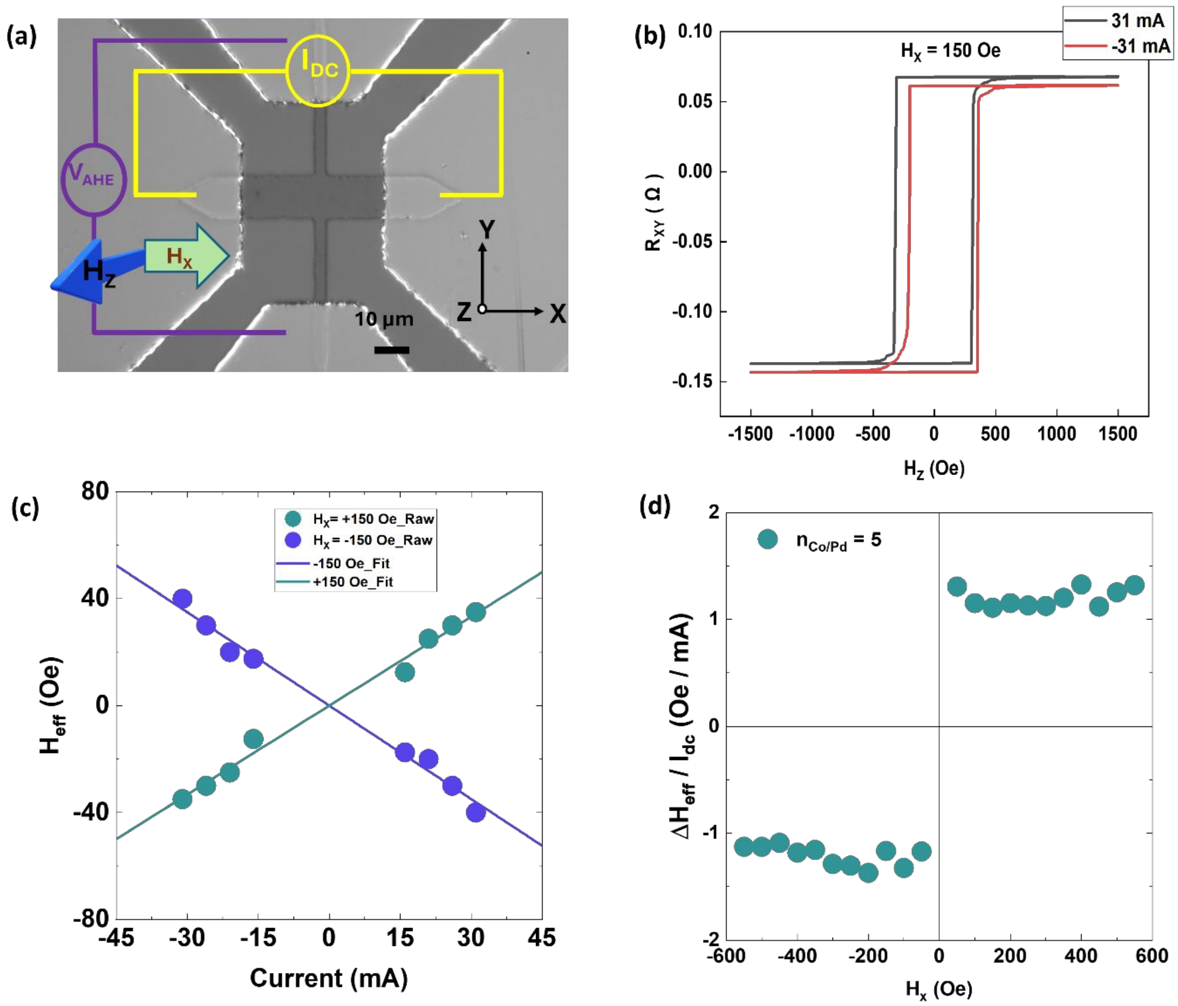

Figure S1: Loop shift measurement. (a) Optical micrograph of the Hall cross device and set up for loop shift measurement, (b) An illustration of loop shift measurement under H_X = 150 Oe for current ± 31 mA, (c) H^Z_{eff}/ I calculation under H_X = 150 Oe, and (d) H^Z_{eff}/ I vs H_X. The saturated value of H^Z_{eff}/ I gives the DL efficiency while the H_X at which H^Z_{eff}/ I is saturated gives H_{DMI} field.

Reference	PtMn composition	FM system	Anisotropy	Metric	Value	Notes
This work	$Pt_{50}Mn_{50}$	$(Co/Pd)_5$ multilayer	PMA	ξ_{DL} (loop-shift)	0.235	Contribution from both PtMn and top Ta.
3	$Pt_{50}Mn_{50}$	Co	In-plane	ξ_{DL}	0.16 ± 0.01	Standard order Ta/PtMn/Co/MgO/Ta.
3	$Pt_{50}Mn_{50}$	Co	In-plane	ξ_{DL}	0.19 ± 0.02	Reverse order (enhanced interface transparency).
3	$Pt_{50}Mn_{50}$	FeCoB (CoFeB)	In-plane	ξ_{DL}	0.096 ± 0.003	Standard order Ta/PtMn/FeCoB/MgO/Ta.
3	$Pt_{50}Mn_{50}$	FeCoB (CoFeB)	In-plane	ξ_{DL}	0.174 ± 0.004	Reverse order.
3	$Pt_{50}Mn_{50}$	FeCoB (CoFeB)	PMA	ξ_{DL}	0.11 ± 0.02	PtMn/FeCoB PMA case.
3	$Pt_{50}Mn_{50}$	FeCoB (CoFeB)	PMA	ξ_{DL}	0.24 ± 0.03	With Hf insertion (interface engineering).
6	$Pt_{38}Mn_{62}$	Co/Ni multilayer	PMA	θ_{SH}	0.19 ± 0.03	Effective-field-based efficiency; composition differs from Pt50Mn50.

Table S1: Comparison of PtMn efficiency in our case with other reports.

In our stack, PtMn is much thicker, and its resistivity is nearly half that of Ta[3]. Using the parallel current shunting method, the fraction of total current flowing into PtMn is 0.3635, while that flowing into the Ta capping layer is 0.0477. Thus, using the parallel current shunting model, we can calculate that the ratio of current flowing into Ta to the current flowing in PtMn is 0.13**.** Thus, the majority of spin current is coming from PtMn during the SOT switching, while Ta, owing to its strong SHE, also contributes to the switching[4]. It has been reported that SOT efficiency can be

enhanced in Pt/Co/Ta due to Ta capping[5]. Because the PtMn has the same SOT sign as Ta, Ta can help the SOT switching[5]. Irrespective of this, the main points of our findings are independent of whether Ta cap helps with SOT current generation, while the observed behavior is coming from the inherent twin defect enhancement with increasing $n_{Co/Pd}$ as observed in the HRTEM analysis, which pins the domain wall motion and changes switching mode from binary to analog for high $n_{Co/Pd}$.

Supplementary Information S2: Effect of pulse width on dual SOT mode in $n_{Co/Pd} \leq 7$.

The pulse width influences both the switching characteristics and the binary-to-analog transition threshold because the reversal dynamics depend on the energy delivered per pulse (Joule heating) and on the time available for domain-wall propagation versus nucleation under a given current density. Thus, we have included these additional measurements demonstrating that the analog/multilevel (dual SOT) behavior is preserved when the pulse width is reduced to 10 μs (tested for representative $n_{Co/Pd}$ values).

Figure S2 shows SOT switching behavior for $n_{Co/Pd} = 3$ in the pulse width ranging from 10 μsec to 300 μsec. We chose $n_{Co/Pd} = 3$ as the current amplitude required for SOT mode switching transition from binary to analog under 10 μsec pulses after large current treatment is within our instrument range. For larger $n_{Co/Pd}$, the required current amplitude under 10 μsec pulses is beyond what the pulse generator can apply. As shown in Figure S2 (a), the SOT switching mode remains binary within the pulse width range of 10 μsec to 300 μsec. As the pulse width increases, the current required for the full binary SOT decreases, a typical SOT switching behavior. As shown in Figure S2 (a) and (b) for 10 μsec pulses, we need about ~ 55 mA current to do the full binary SOT switching, while we need about ~ 93 mA current to do the transition from binary to analog SOT switching mode. For 300 μsec pulses, ~ 45 mA of current is sufficient to switch the magnetization, while about 80 mA is required to induce the transition from binary SOT mode to analog SOT mode, as depicted in Figures S2 (c) and (d), respectively. The difference in the transition currents for 10 μsec and 300 μsec pulses is due to the fact that decreasing pulse width typically requires increasing the current amplitude to surpass the energy threshold to cause twinning defects formation, which pins the domain wall motion, and to have nucleation dominated SOT switching.

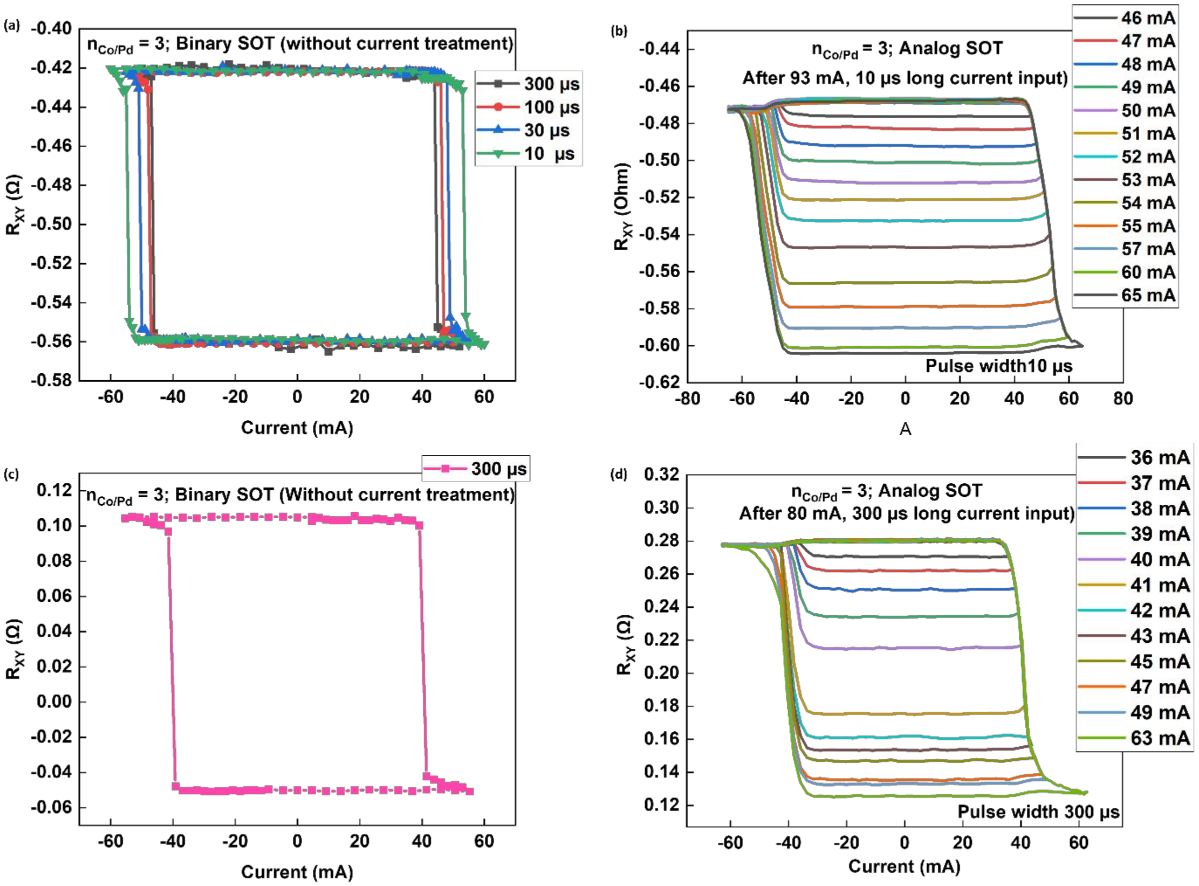

Figure S2: (a) SOT switching behavior in pulse width range of 10 µsec to 300 µsec for $n_{Co/Pd} = 3$. It remained binary in this pulse width range, (b) Analog SOT switching under a pulse width of 10 µsec; the transitioned to an analog one after it was treated with the current of amplitude 93 mA and pulse width of 10 µsec, and (c) Binary SOT switching under 300 µsec pulse width, and (d) Analog SOT switching under a pulse width of 300 µsec; the transitioned to an analog one after it was treated with the current of amplitude 80 mA and pulse width of 300 µsec. All measurements were performed at H_X = 40 Oe.

Supplementary Information S3: Effect of pulse width on SOT mode selection in $\mathbf{n_{Co/Pd}}$.

To verify if the SOT mode selection exists at shorter pulses i.e. depending on $\mathbf{n_{Co/Pd}}$**,** i.e. do low $n_{Co/Pd}$ devices still remained binary like SOT and do high $n_{Co/Pd}$ devices still remained analog like in short pulse regimes, we performed additional SOT switching measurements using 10 µsec pulses (i.e., **30× shorter** than the pulse width used throughout the main manuscript) for representative devices with $n_{Co/Pd} = 3$, $n_{Co/Pd} = 5$, and $n_{Co/Pd} = 8$. These measurement results are shown below in Figure S3. Figure S3 (a) is SOT switching under 10 µsec long for as deposited $n_{Co/Pd} = 3$, S3 (b) is SOT switching under 10 µsec long is for as deposited $n_{Co/Pd} = 5$, and S3 (c) is for is SOT switching under 10 µsec long pulses as deposited $n_{Co/Pd} = 8$.

The results confirm that the key "mode-selection" conclusions remain unchanged under substantially shorter pulses: $n_{Co/Pd} = 3$ and $n_{Co/Pd} = 5$ devices continue to exhibit binary-like switching, whereas the $n_{Co/Pd} = 8$ devices maintained analog/multilevel behavior, with the analog operation achieved at significantly shorter pulses (≈ 30× reduction compared with the 300 µsec long-pulses) for high $n_{Co/Pd}$ while the low $n_{Co/Pd}$ devices still remained binary and transitioned to analog only when subjected to substantially larger current than that required for binary SOT. The $n_{Co/Pd}$ dependent mode selection results under short pulses (10 µsec) is shown below in Figure S3.

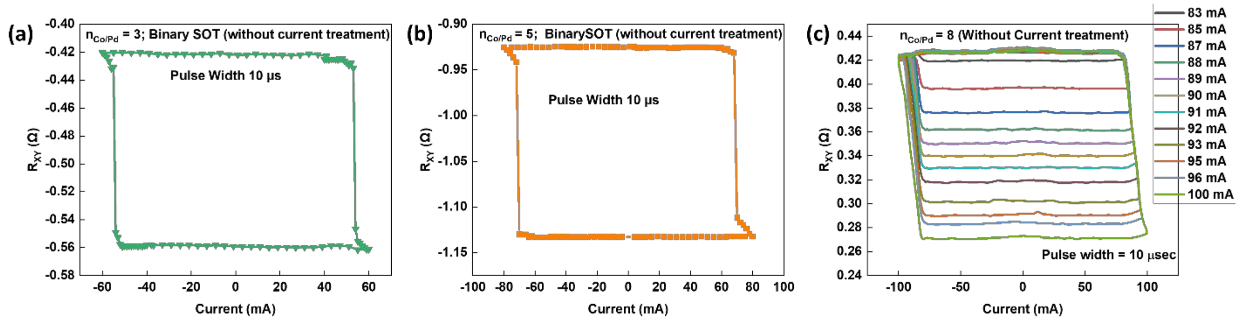

Figure S3: Mode selection at 10 µs pulses. (a) For the as-deposited $n_{Co/Pd} = 3$ device, (b) for the as-deposited $n_{Co/Pd} = 5$ device, and (c) $n_{Co/Pd} = 8$ device. $H_X = 40$ Oe during all the measurements.

Supplementary Information S4: Stability of Hybrid devices and performance consistency of 3 routes to analog SOT

To check the stability and reproducibility of the hybrid-treated devices and to directly evaluate post-treatment robustness under severe operational conditions, we performed an accelerated cycling test on hybrid devices using 100 write pulses (pulse width: 300 μsec) with an amplitude of approximately 95 mA, i.e., the current required to complete the switching. The selected writing pulse amplitudes may cause maximum thermal stress on devices during the switching process. Therefore, we called this test the "thermal stress test". Figure S4 shows robustness of Hybrid devices after maximum thermal stress. As shown in Figure S4 below, the switching portion decreased only slightly (≈3%) after 100 pulses, without any change in current channel resistance (R_{XX}), indicating that the hybrid state is robust against repetitive high-amplitude stressing and does not exhibit rapid degradation.

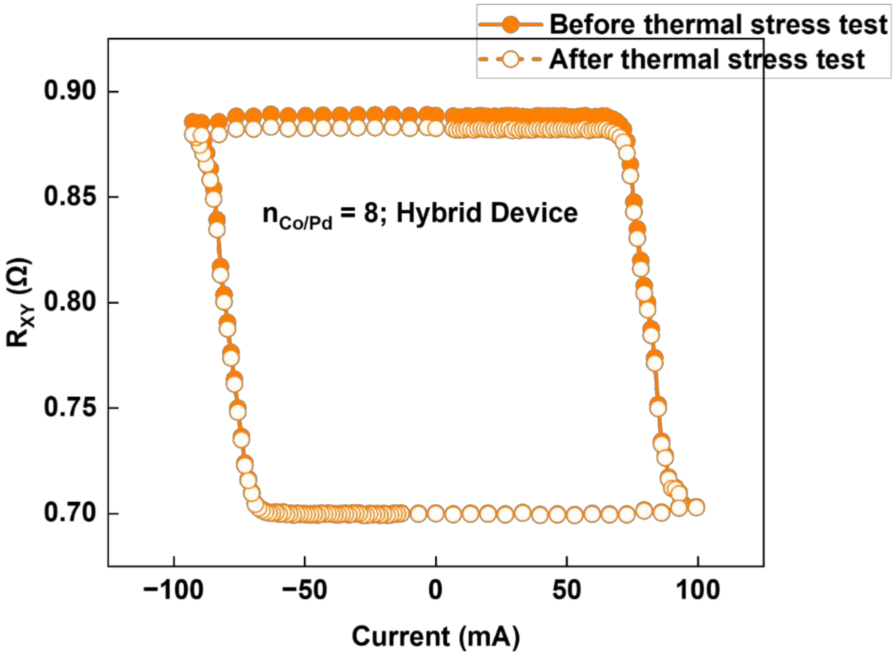

Figure S4: Stability of the Hybrid device after the thermal stress test. The overlapped R_{xy}-current major loops of the devices before and after 100 cycling pulses (thermal stress test). Each data point represents the multilevel states, which are nearly the same. All the SOT switching measurements used H_X = 40 Oe, and current pulses were 300 µsec long.

To further check device reproducibility and robustness, we also repeated the relevant measurements on a device fabricated and deposited in a separate run. The new data set provides several independent and mutually consistent checks that together verify both reproducibility and reliability of hybrid device against thermal stress. Note that, due to target availability, we need to change the electrode (Hall pads) materials for the devices from the original Ti/Pt to Ta/Pt, which changed the as-fabricated device resistance. However, the overall behavior of this newly fabricated

device is the same as that of the previous devices, further confirming the reproducibility of the proposed mechanism.

Figures S5 (a) and (b) are representative R_{XY}–current loops for $n_{Co/Pd}$ = 8 measured in the as-fabricated state (without high current treatment) and after current treatment, respectively. As shown in Figure S5 (a), the SOT switching is analog in the as-fabricated state, i.e., without any high-current treatment. After the device was treated with high current, it switched to hybrid SOT mode with more intermediate states, as shown in Figure S5 (b).

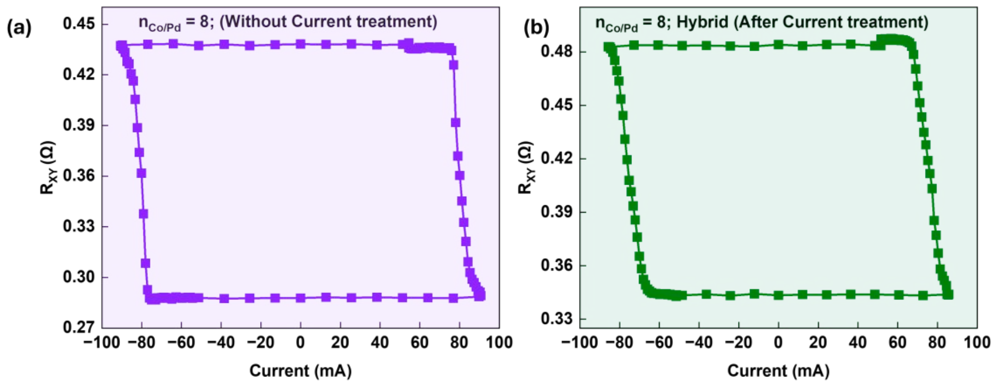

Figure S5: Creation of a Hybrid device using high current treatment of as-deposited $n_{Co/Pd}$ = 8. (a) The as-fabricated $n_{Co/Pd}$ = 8 device is already in an analog state, i.e., before current treatment. (b) SOT curve for $n_{Co/Pd}$ = 8 after treatment (hybrid $n_{Co/Pd}$ = 8 device) with large current; an increase in resistance from 156 Ohm to 165 Ohm, is observed, accompanied by an enhanced number of intermediate states. Each pulse is 300 μsec long with an H_X of 40 Oe.

To directly evaluate device robustness under severe operational conditions, we performed an accelerated cycling test on hybrid devices using 200 write pulses (pulse width: 300 μsec) with an amplitude of approximately ± 86 mA, i.e., the current required to complete the switching of the

hybrid device fabricated in a different run. The measurement and its results are shown in Figure S6. S6 (a) is the applied pulse sequence, and Figure S6 (b) is the result of this thermal stress test under the pulse sequence shown in S6 (a). The variation in switching percentage is about 4 %. Also, after this test, the device's resistance only slightly increased from 165 Ω to 166 Ω (about 0.6 %). This result suggests that the 200-cycle large current pulses only make slight variations in the devices. Compared to the 100-cycle test, the variation in switching percentage is slightly higher, from 3% to 4%, but it remains within an acceptable range.

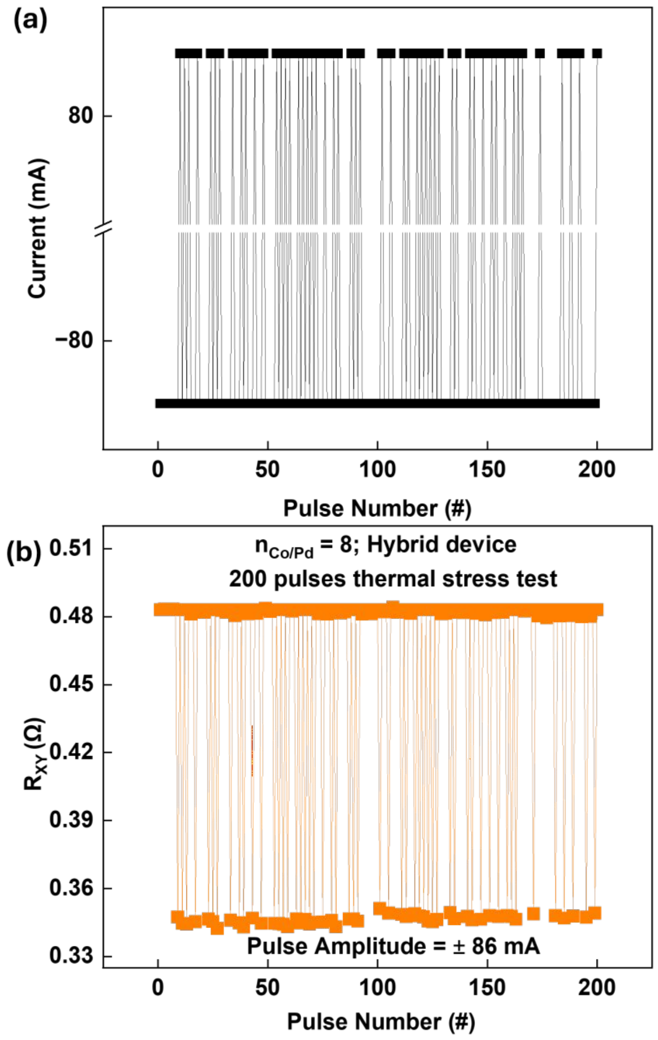

Figure S6: The test with 200 writing cycles, so-called “thermal stress test”, of hybrid $n_{Co/Pd} = 8$ devices. (a) Pulse sequence where we applied pulses with amplitudes of ± 86 mA, required to fully switch magnetization state to $\mp M_Z$; each pulse is followed by a reset pulse of -86 mA to switch the magnetization back to $+M_Z$ before applying the next pulse. The pulse sequence was designed to mimic multiple writing and to maximize thermal stress on devices. (b) Results of thermal stress test. Each pulse is 300 µsec long with an H_X of 40 Oe.

Figure S7 (a) and S7 (b) show the comparison of the hybrid device SOT curves before and after the thermal stress test. Note that the resistance increases slightly by ~0.6% after the thermal stress test. To achieve a similar state, we need to slightly increase the device's writing current amplitude (0.5 - 0.8 %) after thermal stress, except for the initial switching, as shown in Figure S7 (a) and (b) below. Pre- and post-thermal-stress R_{XY}–current loops under multiple pulse amplitudes of 67-86 mA, demonstrate the nearly identical multi-level SOT behavior even after the thermal stress test.

To clearly demonstrate the device’s robustness after the thermal stress test, we further compare the selected intermediate states before and after the test, as shown in Figures S7 (c)–S7 (i). As we can see, the intermediate states for different current amplitudes are also nearly identical, with a slight mismatch near the maximum switchable states. We observed ~4% reduction in the maximum switchable portion before and after the thermal stress test. Compared to the previous 100 cycling test (~3% reduction), our results suggest that thermal stress can induce additional pinning, resulting in slightly reduced switchable portions due to over-pinning. On the other hand, the substantial

overlap before/after the thermal stress is strong evidence of the device's stability after the thermal stress.

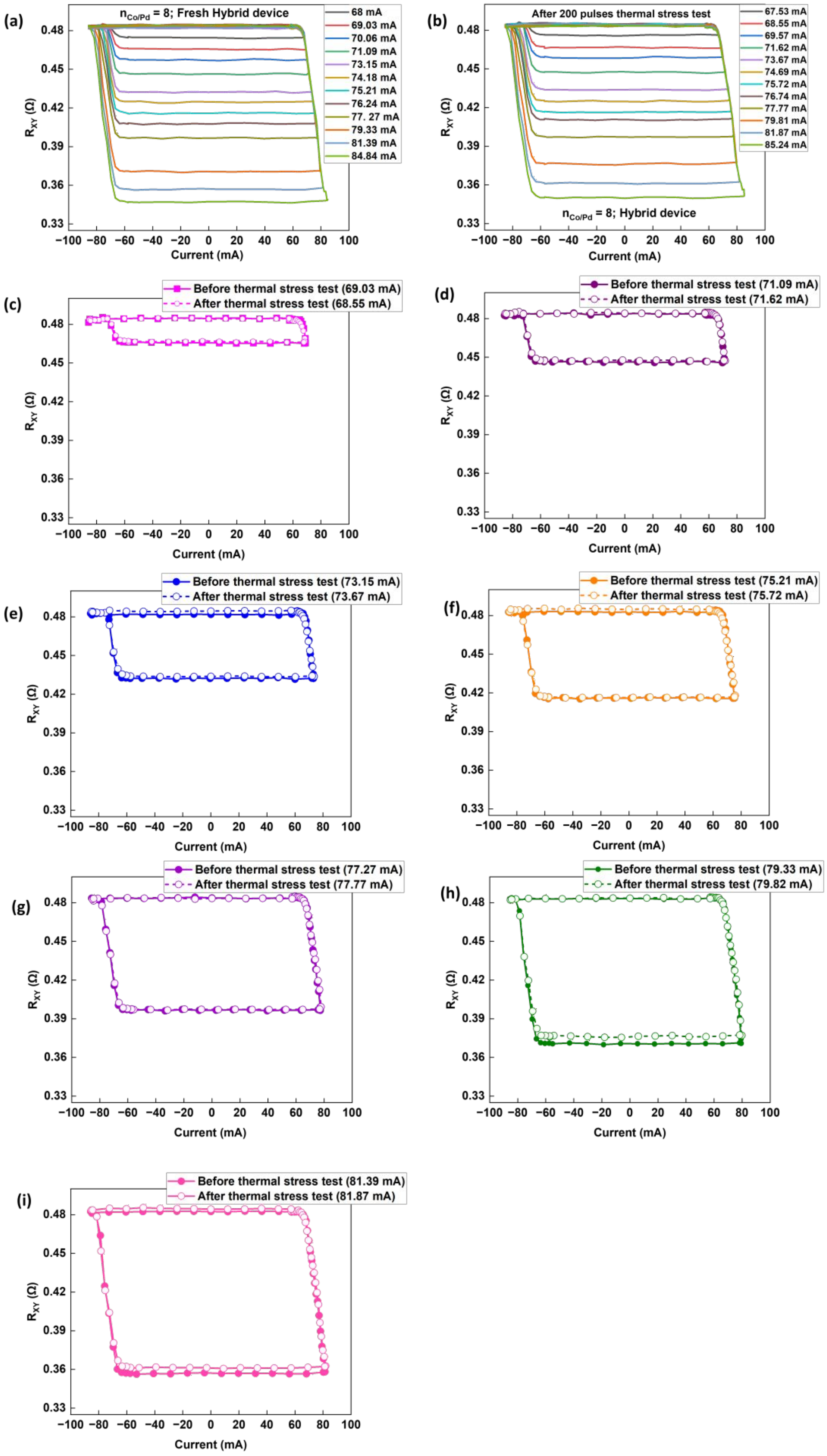

(a)
n$_{Co/Pd}$ = 8; Fresh Hybrid device
68 mA
69.03 mA
70.06 mA
71.09 mA
73.15 mA
74.18 mA
75.21 mA
76.24 mA
77. 27 mA
79.33 mA
81.39 mA
84.84 mA
R$_{XY}$ (Ω)
Current (mA)
(b)
After 200 pulses thermal stress test
67.53 mA
68.55 mA
69.57 mA
71.62 mA
73.67 mA
74.69 mA
75.72 mA
76.74 mA
77.77 mA
79.81 mA
81.87 mA
85.24 mA
n$_{Co/Pd}$ = 8; Hybrid device
R$_{XY}$ (Ω)
Current (mA)
(c)
Before thermal stress test (69.03 mA)
After thermal stress test (68.55 mA)
R$_{XY}$ (Ω)
Current (mA)
(d)
Before thermal stress test (71.09 mA)
After thermal stress test (71.62 mA)
R$_{XY}$ (Ω)
Current (mA)
(e)
Before thermal stress test (73.15 mA)
After thermal stress test (73.67 mA)
R$_{XY}$ (Ω)
Current (mA)
(f)
Before thermal stress test (75.21 mA)
After thermal stress test (75.72 mA)
R$_{XY}$ (Ω)
Current (mA)
(g)
Before thermal stress test (77.27 mA)
After thermal stress test (77.77 mA)
R$_{XY}$ (Ω)
Current (mA)
(h)
Before thermal stress test (79.33 mA)
After thermal stress test (79.82 mA)
R$_{XY}$ (Ω)
Current (mA)
(i)
Before thermal stress test (81.39 mA)
After thermal stress test (81.87 mA)
R$_{XY}$ (Ω)
Current (mA)

Figure S7: Stability of hybrid $n_{Co/Pd} = 8$ devices after thermal stress test (a) Fresh Hybrid device SOT curves, (b) Hybrid device SOT curves after 200 cycles of 86 mA and 300 μsec pulses (thermal stress test), (c-i) A comparison of SOT curves for fresh hybrid device and thermal stress tested hybrid device for different input currents. All the SOT switching measurements used $H_X = 40$ Oe, and current pulses were 300 μsec long.

We note that the hybrid mode relies on the controlled creation of pinning/defect landscapes that stabilize multilevel reversal; therefore, it is essential to minimize unnecessary Joule heating overhead during routine operations. In this regard, we emphasize a practical mitigation approach to further improve endurance margins: the real devices can be made into a pillar structure down to the ferromagnetic layer, while the bottom spin-current generation layer is made into a current channel on which the center pillar ferromagnet is fabricated. The dimensions of the spin-current channel can exceed those of the ferromagnetic pillar. This device design may reduce current shunting in the FM layer and direct most of the current into the spin-current source layer, thereby maximizing SHE efficiency. Thus, the switching current can be reduced compared to typical Hall cross devices like ours, which are mostly used as proof-of-concept to demonstrate the desired functionality, as they are easier to fabricate. Besides, in real SOT devices, the pillars are much smaller, so the total ferromagnetic volume is reduced, further lowering the amplitude and duration of the current required for switching. Thus, by optimizing the device geometry and structure, we can further reduce the switching current and alleviate thermal stress on the ferromagnetic layer due to Joule heating. Our findings are intrinsic to the Co/Pd layers: increasing the repeat number increases defects, leading to analog-like switching, and are independent of the device design and geometry. This demonstrates that the current/thermal load can be meaningfully reduced without

sacrificing the mode-engineering concept. On the other hand, for the hybrid devices, which require a one-time large current treatment, we may need to optimize the device dimension as well as the pulse amplitude and duration of the large current to enable the formation of defects during the high current treatment, but to suppress the thermal effects in the later operation with a small current amplitude and a short duration. This discussion is also included in supplementary information S4 and is referenced in the main manuscript.

To check the robustness of devices showing Dual SOT behavior, i.e., nCo/Pd = 2-7 devices, we tested 10 devices from different sample batches with $n_{Co/Pd}$ = 5 as a representative to determine if devices treated under the same conditions exhibit the same behavior. All the as-fabricated devices exhibited a binary SOT mode with a critical switching current of 70 mA (pulse width = 300 μs), displaying minimal variation in the switching current. All of them transitioned to analog SOT devices when treated with 85 mA, 300 μsec pulses. The device-to-device variations were negligible. The same holds true for $n_{Co/Pd}$ = 8 devices. They are analog SOT and need about 95-96 mA and 300 μsec pulses to complete the multilevel SOT switching. Their multi-levels are further improved if they are treated with a 300 μsec current pulse with an amplitude of ~105 mA, with very little variation among different devices.

Finally, regarding reproducibility, we consistently observed the same hybrid effect in all tested devices fabricated on the same wafer, and we also reproduced this hybrid behavior in devices fabricated in independent deposition runs, confirming that the hybrid mode is not an isolated or device-specific outcome but a reproducible feature of the PtMn/(Co/Pd)n platform under the same conditioning protocol.

Supplementary Information S5: Effect of roughness on mode selection in $n_{Co/Pd}$

To verify if the film's roughness plays a role in the $n_{Co/Pd}$ dependent mode selection, we performed AFM measurements (8 regions per sample) to quantify surface roughness across samples with different repeat numbers. For $n_{Co/Pd}$ = 3, 5, 7, and 9, the measured arithmetic mean roughness Ra is 112.2 pm, 92.3, 118.3 pm, and 89.9 pm, respectively. The representative AFM data for $n_{Co/Pd}$ = 7 are shown in Figure S8 below. These repeated AFM measurements confirmed that although the roughness variations exist, the roughness in all samples is very low. These roughness values indicate that the switching-mode evolution contingent upon increasing $n_{Co/Pd}$ is primarily governed by defect (nucleation-site) density (twin density) increase with $n_{Co/Pd}$ rather than surface roughness. Therefore, we revise the original statement to "Although the roughness for samples with different repeat $n_{Co/Pd}$ may vary, measured arithmetic mean roughness Ra for samples is on the order of 100 pm, a very low level. Therefore, the surface roughness may not be the primary factor affecting the switching mode. The increased repeats with thicker stacks likely accumulate higher defect density, which enhances pinning.

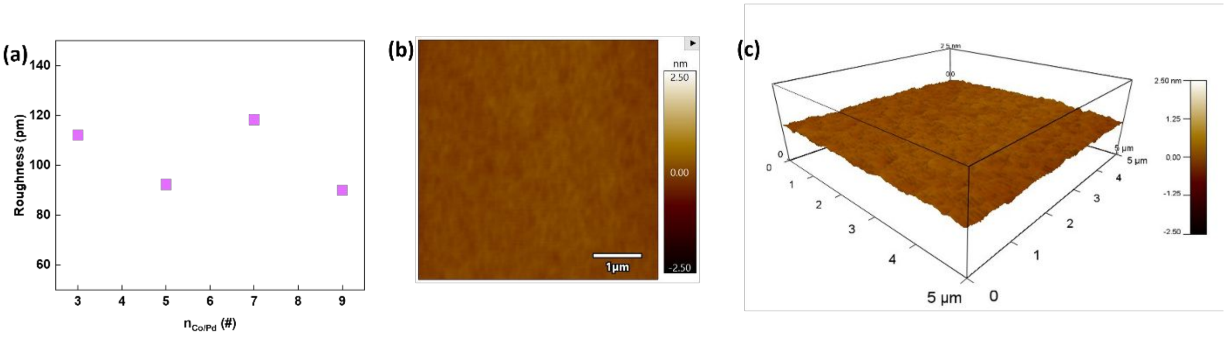

Figure S8: (a) Surface Roughness vs $n_{Co/Pd}$. (b,c) 2D and 3D surface scans for $n_{Co/Pd}$ = 7. These repeated AFM measurements confirmed that although roughness variations exist, the roughness in all samples is very low.

Supplementary Information S6: Difference between Non-Linearity of LTP (NL_{LTP})and Non-Linearity of LTD (NL_{LTD}).

We extract the cycle-to-cycle variations in the linearity of the LTP/LTD characteristics from repeated pulse measurements in Figure 6 (a) – 6 (c). Figures 6 (d) – 6 (f) show the normalized R_{XY} as a function of the normalized pulse number for multiple LTP/LTD cycles, where the data are extracted from Figures 6 (a) – 6 (c). Based on these characteristics, the nonlinearity variations of the potentiation and depression curves across different cycles are quantitatively evaluated and summarized in Figures 6 (g) – 6 (i), respectively. The nonlinearity factors for potentiation (NL_{LTP}) and depression (NL_{LTD}) are calculated using the methodology reported by Chen, Pai-Yu et al.[7]

To clarify the differences between NL_{LTP} and NL_{LTD} we have plotted LTP and LTD for only one cycle for the three cases and shown below in Figure S9 below. It is taken from Figure 6 (a)-Figure 6 (c) in main manuscript. As we can see in Figure S9 (a), for $n_{Co/Pd}$ = 5 (Via current treatment) case, LTP requires 12 pulses, while LTD requires 16 pulses, similarly for $n_{Co/Pd}$ = 8 (without current treatment) case in Figure S9 (b) LTP requires 13 while LTD requires 17 pulses, while for the current treated $n_{Co/Pd}$ = 8 (hybrid case) both LTP and LTD requires 19 pulses each as shown in Figure S9 (c). This difference between the number of pulses requires to fully switch from $-M_Z$ to $+M_Z$ (LTP) and $+M_Z$ to $-M_Z$ (LTD) is the reason behind larger differences in Non-Linearity between LTP and LTD in $n_{Co/Pd}$ = 5 (Via current treatment) and $n_{Co/Pd}$ = 8 (without current treatment) cases. During LTP (Figures S9 (a) – S9 (b), the change in R_{XY} exhibits a nearly constant increase over the entire pulse sequence, resulting in an almost linear potentiation behaviour. In contrast, LTD is linear for the initial 9-10 pulses (Figures S9 (a) – S9 (b)), while for the subsequent 6-7 pulses, the change in R_{XY} with respect to increasing pulse number is smaller, resulting in overall larger non-linear behaviour in LTD.

Since in the Figure 6 (a) and Figure 6 (b) is plotted for 5 cycles, the difference between LTD and LTP visually seems less pronounced due to expanded x-axis but in reality the calculated values of NL_{LTP} and NL_{LTD} is different due to different number of pulses needed to realize LTP and LTD which is more apparent in Figures 6 (g) and (h). On the other hand for the hybrid $n_{Co/Pd} = 8$ (after current treatment) case in Figure 6 (c) of the main manuscript or Figure S9 (c) below, LTP requires 19 and LTD also requires 19 pulses each, thus we can clearly see the difference between calculated values of NL_{LTP} and NL_{LTD} is less in Figure 6 (i) in the main manuscript.

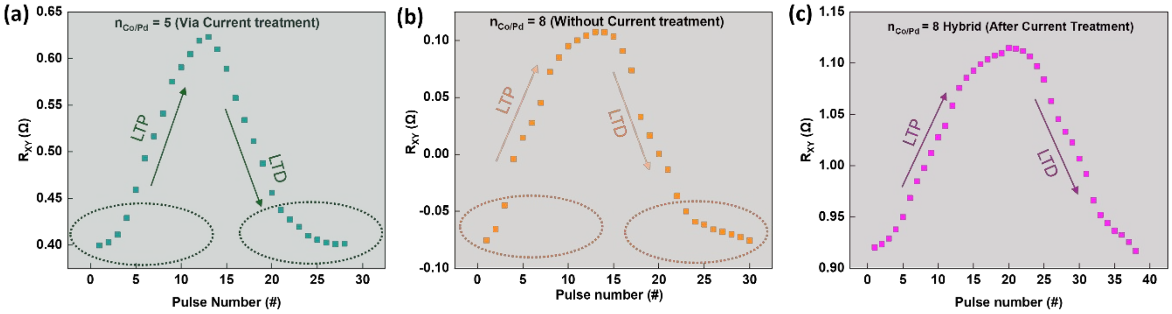

Figure S9: Synaptic plasticity performance for three analog SOT devices. LTP/LTD curve depicting the non-linearity, and asymmetry of synaptic weights update for 1 cycle only (a) $n_{Co/Pd} = 5$ with a large current treatment, (b) $n_{Co/Pd} = 8$ without current treatment, (c) $n_{Co/Pd} = 8$ (Hybrid, after current treatment).

References:

(1) Hu, C.-Y.; Chen, W.-D.; Liu, Y.-T.; Huang, C.-C.; Pai, C.-F. The Central Role of Tilted Anisotropy for Field-Free Spin–Orbit Torque Switching of Perpendicular Magnetization. *NPG Asia Mater.* 2024, *16*, 1. DOI: 10.1038/s41427-023-00521-9.

(2) Pai, C.-F.; Mann, M.; Tan, A. J.; Beach, G. S. D. Determination of Spin Torque Efficiencies in Heterostructures with Perpendicular Magnetic Anisotropy. *Phys. Rev. B* 2016, *93* (14), 144409.

(3) Pham, N. L. L.; Ko, K.-H.; Choi, G.-M. Ferromagnetic Material Dependence of Spin–Orbit Torque in PtMn/Ferromagnet Bilayer. *Appl. Phys. Lett.* 2023, *123* (16), 162402.

(4) Liu, L.; Pai, C.-F.; Li, Y.; Tseng, H.-W.; Ralph, D. C.; Buhrman, R. A. Spin-Torque Switching with the Giant Spin Hall Effect of Tantalum. *Science* 2012**,** *336* (6081), 555–558.

(5) Woo, S.; Mann, M.; Tan, A. J.; Caretta, L.; Beach, G. S. D. Enhanced Spin-Orbit Torques in Pt/Co/Ta Heterostructures. *Appl. Phys. Lett.* 2014, *105* (21), 212404.

(6) Ou, Y.; Shi, S.; Ralph, D. C.; Buhrman, R. A. Strong Spin Hall Effect in the Antiferromagnet PtMn. *Phys. Rev. B* **2016,** *93* (22), 220405.

(7) Chen, P. Y.; Peng, X.; Yu, S. NeuroSim: A circuit-level macro model for benchmarking neuro-inspired architectures in online learning. *IEEE Trans. Comput. Des. Integr. Circuit Syst.* 2018. *37*(12), 3067-3080.
